# *De novo* transcriptome characterization and gene expression profiling of the desiccation tolerant moss *Bryum argenteum* following rehydration

**DOI:** 10.1186/s12864-015-1633-y

**Published:** 2015-05-28

**Authors:** Bei Gao, Daoyuan Zhang, Xiaoshuang Li, Honglan Yang, Yuanming Zhang, Andrew J. Wood

**Affiliations:** Key Laboratory of Biogeography and Bioresource in Arid Land, Xinjiang Institute of Ecology and Geography, Chinese Academy of Sciences, Urumqi, 830011 China; University of Chinese Academy of Sciences, Beijing, 100049 China; Department of Plant Biology, Southern Illinois University-Carbondale, Carbondale, IL 62901-6509 USA

**Keywords:** Transcriptome, Gene expression, Desiccation, *Bryum*, *Physcomitrella*, Biological soil crust

## Abstract

**Background:**

The desiccation-tolerant moss *Bryum argenteum* is an important component of the Biological Soil Crusts (BSCs) found in the Gurbantunggut desert. Desiccation tolerance is defined as the ability to revive from the air dried state. To elucidate the molecular mechanisms related to desiccation tolerance, we employed RNA-Seq and digital gene expression (DGE) technologies to study the genome-wide expression profiles of the dehydration and rehydration processes in this important desert plant.

**Results:**

We applied a two-step approach to investigate the gene expression profile upon rehydration in the moss *Bryum argenteum* using Illumina HiSeq2000 sequencing platform. First, a total of 57,247 transcript assembly contigs (TACs) were obtained from 54.79 million reads by *de novo* assembly, with an average length of 863 bp and N50 of 1,372 bp. Among the reconstructed TACs, 36,916 (64.5 %) revealed similarity with existing protein sequences in the public databases. 23,509 and 21,607 TACs were assigned GO and KEGG annotation information, respectively. Second, samples were taken from 3 hydration stages: desiccated (Dry), rehydrated 2 h (R2) and rehydrated 24 h (R24), and DEG libraries were constructed for Differentially Expressed Genes (DEGs) discovery. 4,081 and 6,709 DEGs were identified in R2 and R24, compared with Dry, respectively. Compared to the desiccated sample, up-regulated genes after two hours of hydration are primarily related to stress responses. GO function enrichment network, EKGG metabolic pathway and MapMan analysis supports the idea of the rapid recovery of photosynthesis after 24 h of rehydration. We identified 770 transcription factors (TFs) which were classified into 50 TF families. 142 TF transcripts were up-regulated upon rehydration including 23 members of the ERF family.

**Conclusions:**

In this study, we constructed a pioneering, high-quality reference transcriptome in *B. argenteum* and generated three DGE libraries to elucidate the changes of gene expression upon rehydration. Expression profiles consistent with the rapid recovery of photosynthesis (at R2) and the re-establishment of a positive carbon balance following rehydration (at R24) were observed. Our study will extend our knowledge of bryophyte transcriptomes and provide further insight into the molecular mechanisms related to rehydration and desiccation-tolerance.

**Electronic supplementary material:**

The online version of this article (doi:10.1186/s12864-015-1633-y) contains supplementary material, which is available to authorized users.

## Background

Desiccation tolerance (DT) is characteristic of many organisms [1, 2]. DT is a common feature of the land plant life cycle and is often restricted to reproductive structures such as seeds and spores [3, 4]. DT of vegetative tissues such as leaves is rare in angiosperms [3] and common in mosses [5]. In many arid and semiarid ecosystems, vegetative cover is sparse. The open spaces in such areas are covered by desiccation-tolerant mosses, cyanobacteria, fungi, green algae and lichens, which are the main components of biological soil crusts (BSCs) [6, 7]. In arid regions, BSCs play a number of important ecological roles which include reducing soil erosion, improving water infiltration and contributing to carbon assimilation and nitrogen fixation [6, 7]. The Gurbantunggut desert (Xinjiang, China) is one of the major arid regions of central Asia and is the largest fixed and semi-fixed cold desert in China. BSCs of the Gurbantunggut desert are dominated by two moss species: *Syntrichia caninervis* and *Bryum argenteum* [7]. BSCs of the Tengger desert (found in northwest China) are dominated by *B. argenteum* [8, 9]. In the desert environment, moss crusts are frequently subjected to cycles of dehydration, desiccation and rehydration [10]. Organisms found in BSCs have developed a suite of adaptive mechanisms that permit the avoidance of water loss and/or the survival of complete dehydration (i.e. desiccation) [10, 11].

More than 200 moss species have been experimentally verified to be desiccation-tolerant and *Tortula ruralis* (= *Syntrichia ruralis*) is the model species for understanding the molecular aspects of vegetative desiccation-tolerance in mosses [1, 2, 4]. *B. argenteum*, like *S. caninervis* and *T. ruralis*, is classified as the most desiccation-tolerant moss species, able to survive equilibration with extremely dry air (i.e. 0-30 % RH) which could tolerant to 30 % RH or below [5]. A common feature of desiccation-tolerant mosses is the rapid recovery of photosynthesis and the rapid re-establishment of a positive carbon balance following rehydration [12]. Previous study suggested that on rehydration of the dry bryophytes, both the photosynthetic electron flow and the PSII activity recovered quickly in *B. argenteum* [13]. Similar to other desiccation-tolerant mosses, recent studies suggested that the thylakoid protein complexes of *B. argenteum* degrade during dehydration and then reassemble following rehydration, and synthesis of proteins, at least those involved in thylakoid protein complexes, was required for the full recovery of photosynthesis after rehydration [11].

In this study, we employed next generation Illumina sequencing technology to investigate the *B. argenteum* transcriptome and conduct comparisons of the digital gene expression profiles. This study was performed based on a two-step approach: (a) Generating and annotating the integrated transcriptome of *B. argenteum*, and (b) Constructing digital gene expression (DGE) libraries to identify differentially expressed genes (DEGs) between the desiccated (Dry) and subsequently rehydrated (R2 and R24) gametophytes. To our knowledge this is the first description of the *B. argenteum* transcriptome and one of the few annotated bryophyte transcriptomes. Our study extends our knowledge of bryophyte transcriptomes and provides further insight into the molecular mechanisms related to rehydration and desiccation-tolerance.

## Results and discussion

### Transcriptome sequencing and *de novo* assembly

In the absence of a reference genome for *Bryum argenteum*, we assembled a *de novo* reference transcriptome for reads mapping and gene expression profiling. To obtain a comprehensive transcriptome in *B. argenteum* and an overview of its gene expression profiles at various dehydrated/rehydrated stages, an equal mixture of total RNAs isolated from various dehydration and rehydration time points were used to construct the sequencing library. Gametophores were harvested from three different time points: desiccated (Dry), rehydrated for 2 h (R2) and rehydrated for 24 h (R24). Using the Illumina NGS platform HiSeq2000, approximately 62.11 million raw sequencing reads were obtained, and 54.79 million paired 90 bp clean reads were obtained after adapter and low-quality reads trimming.

**Fig. 1:**
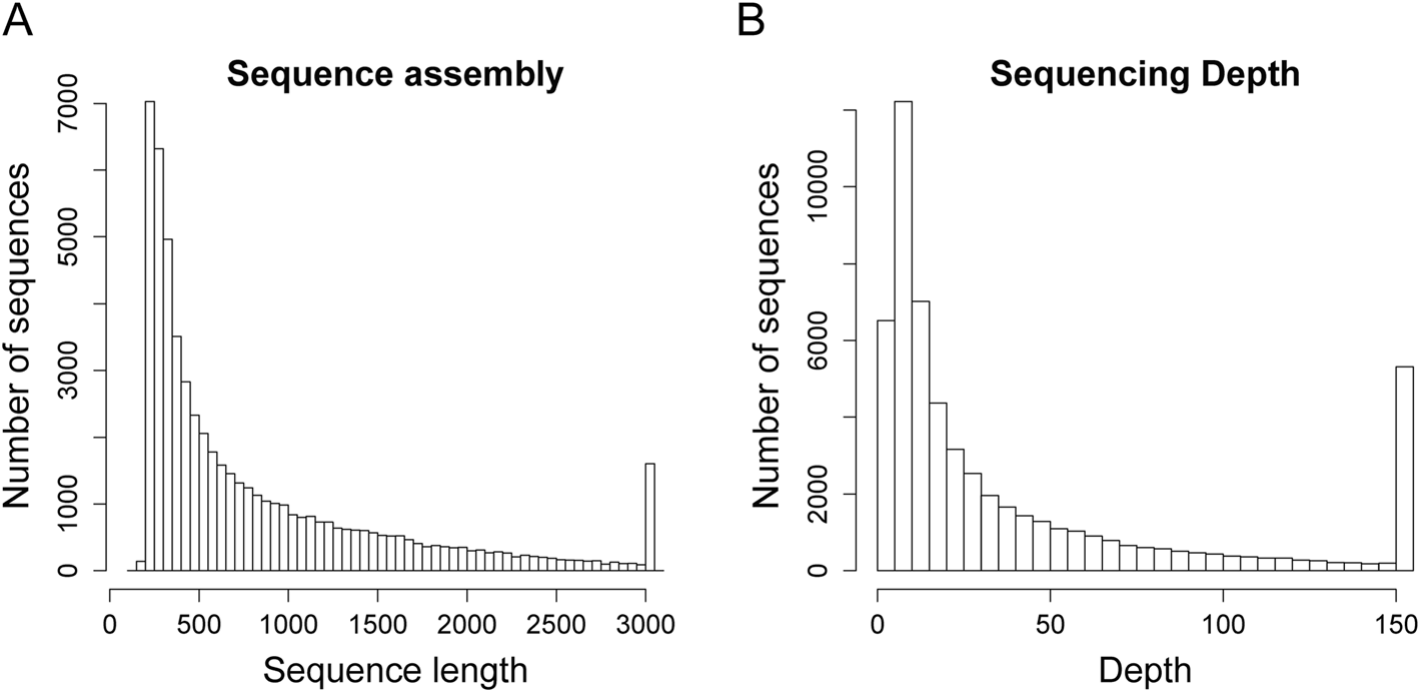
Overview of the *B. agenteum* transcriptome sequencing and assembly. Histogram of the length (**a**) and sequencing depth (**b**) of transcript assembled contigs

With the Trinity *de novo* assembler 106,066 contigs were produced, including different isoforms per contig. To obtain all non-redundant consensus transcript sequences, these initially assembled contigs were clustered using the TGICL to generate the final transcript assembly contigs (TACs hereafter). The statistical characteristics of these contigs and final TACs were shown in Table 1. All reconstructed TACs longer than or equal to 200 bp were retained for further analysis. A total of 57,247 TACs were represented by 14,108 distinct clusters and 43,139 singletons. The longest TAC has a length of 14,247 bp. The final retained TAC set has an average length of 863 bp and N50 of 1,372 bp. The abundance of TACs were evaluated relative to length (Fig. 1a) and sequencing depth (Fig. 1b), respectively. An average sequencing depth of 85× for the final transcriptome assembly was achieved. We then used the *P. patens* reference genome [14, 15] and the ortholog hit ratio method [16] to evaluate the *B. argenteum* TAC integrity, the result indicated that more than 95 % of the putative *P. patens* orthologs captured within the *B. argenteum* transcriptome covered at least 90 % of the predicted ORF (Additional file 1: Figure S1). The 357 *Arabidopsis thaliana* single copy genes conserved across all eukaryotes (UCOS, see Methods) was used to further assess the completeness of transcriptome. As a result, the *B. argenteum* transcriptome assembly contained 352 putative homologs of the 357 *Arabidopsis thaliana* UCOS proteins. And similar results were also obtained for the newly sequenced *Ceratodon purpureus* transcriptome [17]. This suggested that we have assembled and captured an intact transcriptome with respect to transcript integrity and trancriptome completeness. Based upon these metrics, this *B. argenteum* transcriptome is of higher quality than the *S. caninervis* transcriptome reported previously by our group [18].

**Table 1:** Summary of the B. argenteum transcriptome assembly and annotation

Reads	Total Raw Reads	62,112,418
	Total Clean Reads	54,787,000
	Clean Nucleotides (nt)	4,930,830,000
	Reads length (nt)	90 × 2
	Q20 percentage	98.67 %
	GC percentage	52.05 %
Contigs	Total Number	106,066
	Total Length (nt)	43,542,237
	Mean Length (nt)	411
	N50 (nt)	908
Unigenes	Total Number	57,247
	Distinct Clusters	14,108
	Distinct Singletons	43,139
	Total Length (nt)	49,383,291
	Mean Length (nt)	863
	N50 (nt)	1,372
	L50 number	11,094
	Min Unigene Length (nt)	200
	Max Unigene Length (nt)	14,247
	Average Depth (×)	85.38
Annotation	NCBI-nr	35,813 (62.6 %)
	Swiss-Prot	20,864 (39.9 %)
	KEGG	21,607 (37.7 %)
	COG	16,715 (29.2 %)
	GO	23,509 (41.1 %)
	All	36,446 (63.7 %)

### Functional annotation of *B. argenteum* transcriptome

To identify the putative functions of TACs related to the rehydration process, a sequence similarity search was carried out against protein sequences available at NCBI-nr, Swiss-Prot, KEGG and COG databases using BLASTX algorithm with an E-value threshold of 1e-5 (Additional file 2). Among the 57,247 reconstructed TACs, similarity with known proteins was detected for 36,446 TACs which represent 64 % of the *de novo* reference transcriptome assembly (Table 1 and Additional file 1: Figure S2). Among the TACs that returned a positive BLASTX hit within the NCBI-nr database, more than 70 % of the best aligned sequences were shown to be derived from the model moss *P. patens* (Additional file 1: Figure S3). Similar results have been depicted for the desert moss *S. caninervis* [18]. Many TACs could not be matched to a protein sequence within the databases and therefore lack a functional description. Lack of sequence identity with known proteins is partially explained by the limited genomic data available for mosses. To date, the *P. patens* genome is the only moss reference genome [14]. Unmatched sequences could be orphan genes, non-coding RNA or sequences from UTR regions, and we cannot exclude the possibility of partially or misassembled transcripts.

**Fig. 2:**
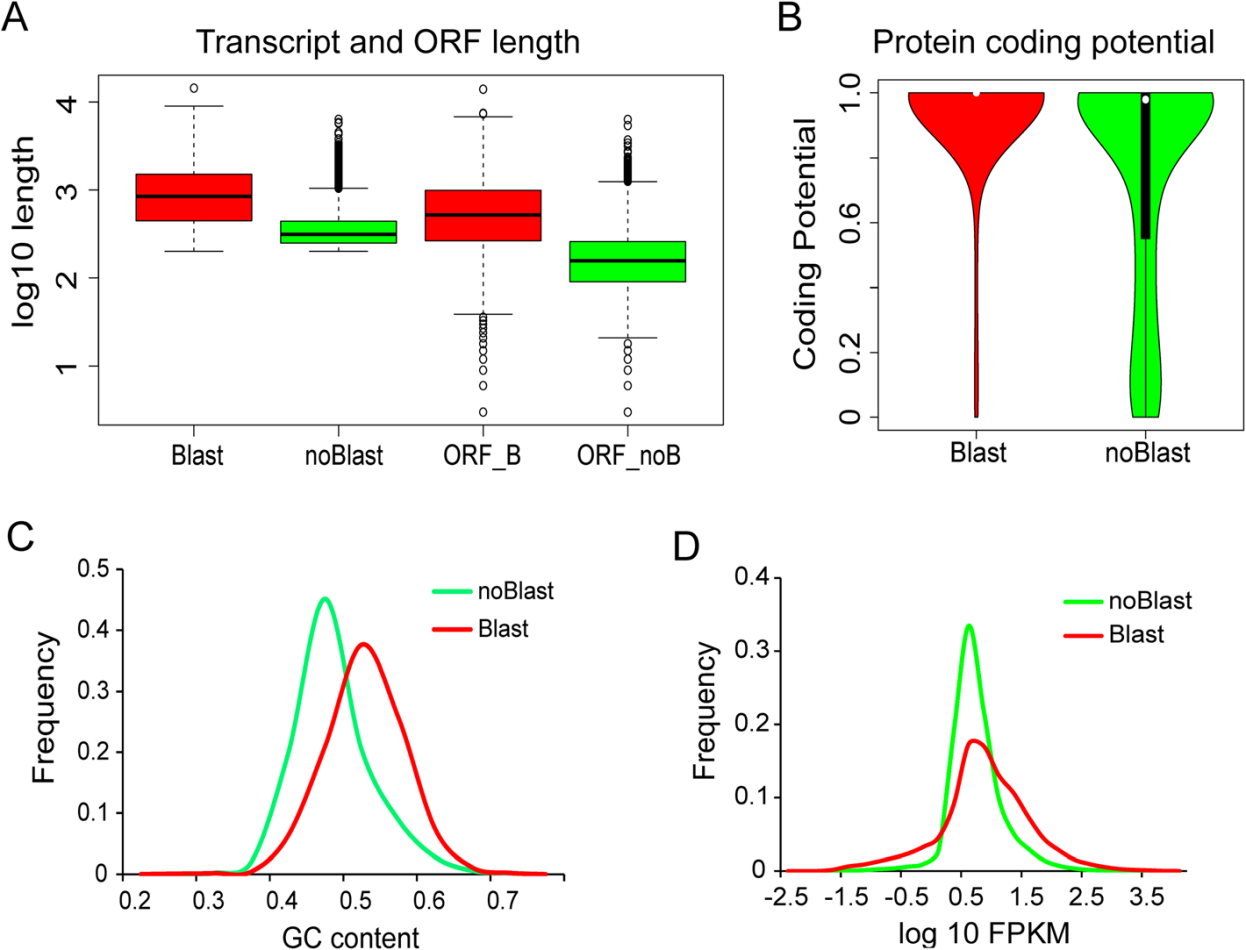
Sequence and expression characterization of the transcripts with and without detected homologs. (**a**) Overall length and ORF length, (**b**) Protein coding potential determined by CPAT, (**c**) distribution of GC content, (**d**) Overall normalized expression level

For further descriptive analysis, we divided our transcriptome TAC set into two subsets: TACs with detected protein homology (‘with BLAST’) and TACs without protein homology (‘without BLAST’). The length of longest open reading frames (ORF) and the protein coding potential score of all TACs were determined by CPAT software [19]. Boxplot of the TACs and ORF length of the two sets is depicted in Fig. 2a. The ‘with BLAST’ group (average length = 1,124 bp) had larger TAC sizes than the ‘without BLAST’ (average length = 405 bp), and same to the ORF sizes (mean length of ‘with BLAST’ and ‘without BLAST’ groups were 730 bp and 209 bp, respectively). Furthermore, higher protein coding potential score, higher GC content and higher expression levels were all encountered for the ‘with BLAST’ group (Fig. 2b-d). Results similar to those obtained for the ‘with BLAST’ have been reported for the wasp, which is also a species without a reference genome [20]. The characteristics of TACs lacking homology - shorter ORFs, lower protein coding potential, lower GC content and lower expression values - may suggest that a large proportion of novel TACs correspond to non-protein coding transcripts [21]. But low sequence conservation across species, lacking of genomic information for desert mosses and the typical tissue specificity [21] of the non-coding RNAs may also explain the novelty of these genes and prevent their annotation.

**Figure 3:**
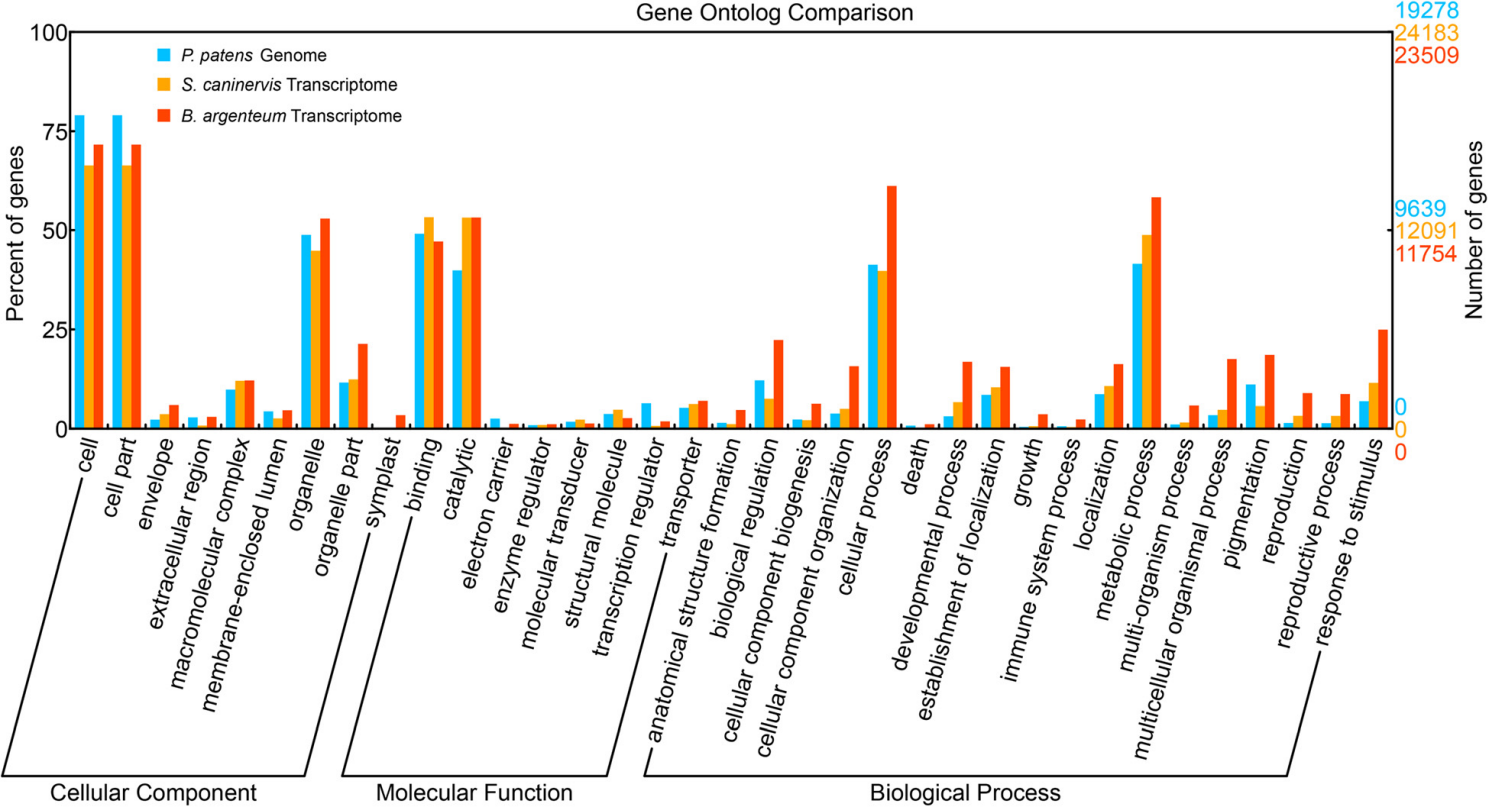
Gene Ontology classification of the *B. argenteum* transcriptome and comparison with the mosses *P. patens* and *S. caninervis*. Gene ontology annotation results of the genes from the *P. patens* genome, *S. caninervis* and *B. argenteum* transcriptome were mapped to categories within the second level of GO terms. GO terms that contain more than 1 % of total genes were included in this graph

To understand the putative functional categories of the annotated TACs, gene ontology (GO) was employed to classify the annotated transcripts. Of the 36,466 TACs with significant similarity to NCBI-nr proteins, 23,509 (64 %) were assigned to GO terms (Fig. 3) and the widely used gene Clusters of Orthologous Groups (COG) functional classification result was illustrated in Additional file 1: Table S1. Previous GO annotation in the mosses *S. caninervis* and *P. patens* assigned a functional annotation of 48 % [18] and 58 % [15], respectively. GO comparison between *S. caninervis*, *P. patens* and *B. argenteum* demonstrated similar sequence enrichment across all three GO categories and is consistent with previous results [18]. For all three moss species transcripts were enriched within the Cellular Component category (“cell”, “cell part” and “organelle”), the Molecular Function category (“binding” and “catalytic”) and the Biological Processes category (“cellular process” and “metabolic processes”). GO functional categories such as “symplast”, “biological regulation”, “cellular component organization”, “cellular processes”, “developmental processes”, “multicellular organismal process” and “response to stimulus” are significantly more represented in *B. argenteum* as compared to the other two mosses.

Kyoto Encyclopaedia of Genes and Genomes (KEGG) provides a basic platform for the systematic analysis of gene function in terms of the metabolic networks of gene products [22]. For further understanding of the biological functions and interactions of genes, pathway-based analysis was conducted based on the KEGG Pathway database. TACs annotated by KEGG orthology proteins were mapped to the metabolic pathways. A total of 21,607 TACs were mapped to 127 reference canonical pathways. The annotations and classifications for these TACs provided a resource for investigating specific pathways in *B. argenteum*, such as photosynthetic carbon reduction and cell wall biosynthesis.

Identifying conserved domains present within a deduced polypeptide can provide insight into the function, regulation and/or localization of the predicted protein. The deduced polypeptide sequences from the *B. argenteum* transcriptome were queried for the presence of protein motifs using the Pfam database [23]. 23,654 deduced polypeptide sequences were assigned Pfam domain/family information and categorized into 4,091 Pfam families (Additional file 3). Among the sequences with a detectable Pfam domain, 22,600 (95.5 %) sequences had homology sequences (‘with BLAST’) in public protein databases, and 1,054 (4.5 %) sequences had no BLASTX hits (‘without BLAST’) but protein domains could be detected using HMMER. Then Pfam domains/families were ranked according to the frequency of occurrence of *B. argenteum* TACs. And among the identified protein domains/families, ‘Pkinase’ and its subclass ‘Pkinase-Tyr’, ‘WD40’, ‘RRM_1’ and ‘ABC-tran’ were the most abundant ones (Additional file 3), similar results was also reported for the *S. caninervis* transcriptome [18].

Transcription factors (TFs) play key roles in plant development and stress response by temporarily and spatially regulating the transcription of their target genes. In this study, we conducted a comprehensive annotation and classification of the TFs in *B. argenteum*. A total of 770 TACs were identified and classified into 50 TF families (Additional file 4) according to the family assignment rules illustrated in PlantTFDB v3.0 [24]. ERF and bHLH TF families revealed to be the two largest TF families, consisting of 91 and 63 gene family members, respectively.

### Expression analysis and identification of differentially expressed genes

**Fig. 4:**
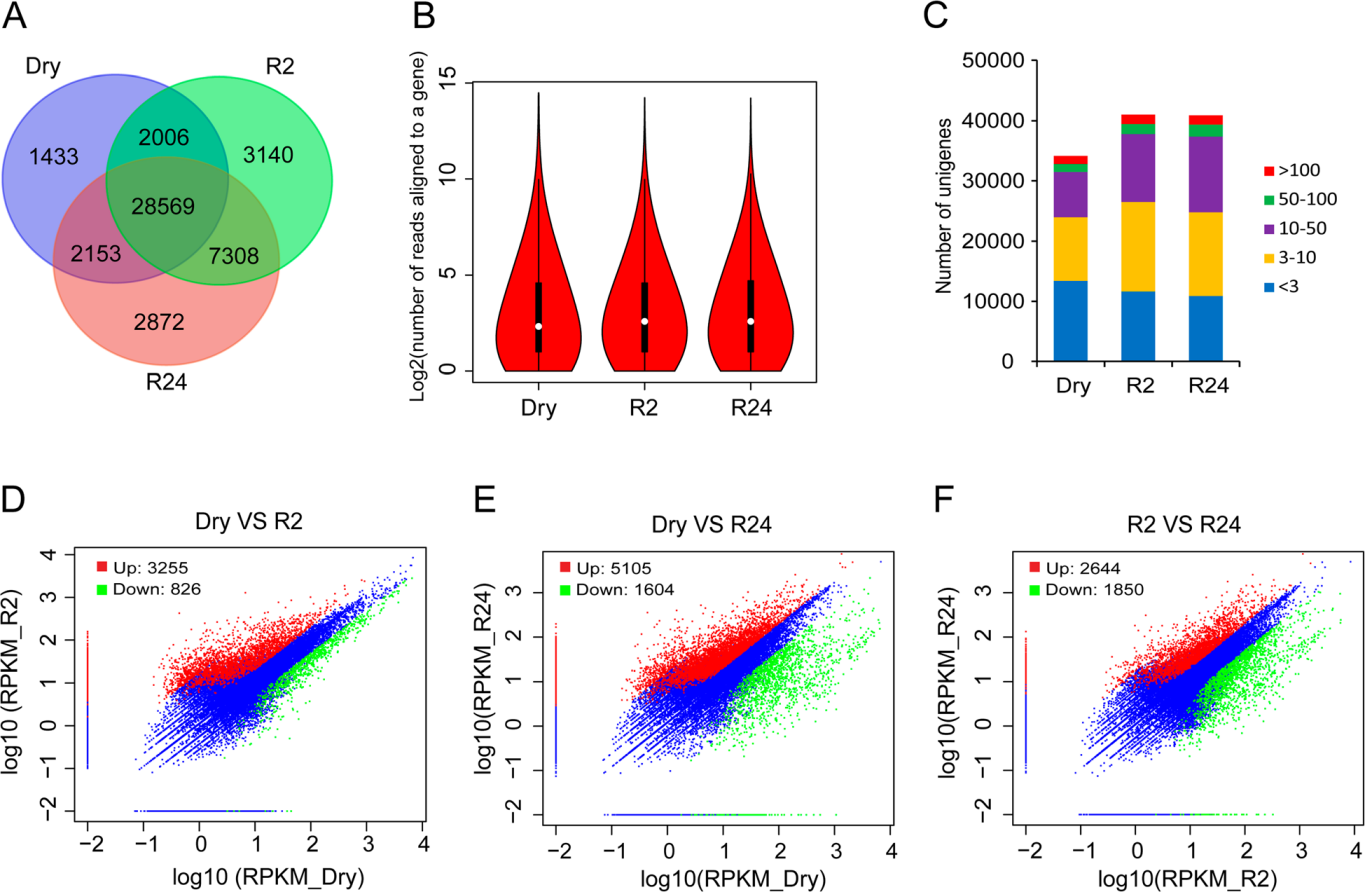
Summary of DGE-seq mapping data and comparison of expressed transcripts between dehydrated and rehydrated samples. **a** Numbers of shared and unique TACs among dehydrated and rehydrated gametophores. **b** Violin plot for the number of reads uniquely mapped to a TAC. **c** Number of transcripts with different expression levels in dehydrated and rehydrated gametophores as measured by DGE sequencing. **d**-**f** The scatter plot comparing the gene expression levels pairwise among the three libraries (between Dry and R2, between Dry and R24, as well as between R2 and R24, respectively). The number of DEGs were also present in the figure

To investigate the digital gene expression profiles of the desiccated and rehydrated gametophores, three digital gene expression (DGE) libraries (Dry, R2 and R24, see Methods) were constructed and sequenced using Illumina deep sequencing technology. More than 3.4 million clean reads were obtained from each library and then aligned to the reference transcriptome separately. Approximately, 58 % (Dry), 55 % (R2) and 50 % (R24) reads mapped uniquely to the reference transcriptome assembly, and 15-19 % of reads were filtered as multiple-aligned and ignored in subsequent analyses (Table 2). The three DGE libraries shared a total of 28,569 TACs, accounting for 50 % of the reference transcriptome assembly, and 1,433 (Dry), 3140 (R2) and 2,872 (R24) TACs were putatively uniquely expressed. A comparison between Dry and the rehydrated DGE libraries (R2 and R24) indicated that 96 % of genes detected in the Dry library were also detected in either one or both of the rehydrated libraries (Fig. 4a). The number of reads aligned to a TAC ranged from 1 to 22,846, with a mean of 62.2 for Dry, 46.5 for R2 and 42.9 for R24. Violin plots for the number of reads aligned to a TAC are depicted in Fig. 4b. Based on the number of uniquely aligned reads, each TAC was assigned with a RPKM value [25], which could normalize for the total reads obtained in each individual library (Additional file 5). Based on this analysis, the gene expression levels in desiccated and rehydrated libraries were classified into five categories (rare, low, moderate, high and extremely high) (Fig. 4c). The largest portion of TACs in Dry exhibited rare expression (RPKM < 3). However, the low expressed TACs (RPKM > 3–10) occupied the biggest proportion in the rehydrated libraries. Only a small fraction (3.7 % to 3.9 %) of TACs was expressed at extremely high levels (RPKM > 100). The average RPKM values of transcripts for Dry, R2 and R24 were 27.3, 24.1 and 23.8, respectively. Furthermore, functional classification of GO terms of the TACs detected from Dry, R2 and R24 were shown in Additional file 1: Figure S4.

**Table 2:** Summary for DGE sequencing datasets

Summary	Dry	R2	R24
Total clean reads	3,666,367	3,488,408	3,488,106
Aligned reads (N)	2,824,311	2,453,703	2,278,641
Aligned reads (%)	77.03 %	70.34 %	65.33 %
Perfect aligned reads (N)	2,500,618	2,150,062	2,000,264
Perfect aligned reads (%)	68.20 %	61.63 %	57.35 %
Uniquely aligned reads (N)	2,125,713	1,908,260	1,756,516
Uniquely aligned reads (%)	57.98 %	54.70 %	50.36 %
Unigenes with uniquely aligned reads (N)	34,161	41,023	40,902
Unigenes with uniquely aligned reads (%)	59.67 %	71.66 %	71.45 %

Differentially expressed genes (DEGs) were determined by applying the screening thresholds of 2-fold changes and FDR ≤ 0.001 [26]. Based on this analysis, the number of up-regulated DEGs were 3,255, 5,105 and 2,644, and down-regulated DEGs were 826, 1,604 and 1,850 in ‘Dry v R2’, ‘Dry v R24’ and ‘R2 v R24’, respectively (Fig. 4d-f). The ranking of up-regulated DEGs (from lowest to highest) is ‘R2 v R24’, ‘Dry v R2’ and ‘Dry v R24’ while the ranking of down-regulated DEGs is ‘Dry v R2’, ‘Dry v R24’ and ‘R2 v R24’. Interestingly, “Dry v R24’ had the most up-regulated DEGs while ‘R2 v R24’ had the most down-regulated DEGs. These results suggest that the rehydration process is associated with the accumulation of novel transcripts, and that some transcripts are more abundant immediately after rehydration (i.e. R2) and are associated with an ‘early response” to rehydration. All DEGs which exceed the threshold are listed in the Additional file 5.

Since TFs play crucial roles in the regulation of gene expression and plant stress response, we performed comprehensive analysis of the differentially expressed TFs. All annotated and differentially expressed *B. argenteum* TFs were listed in Additional file 4. The most up-regulated TF after 2 h of rehydration was a MYB_related transcription factor (unigene20865_TBA), which contains a specific Myb_dna_bind domain (PF00249). The most up-regulated TF after 24 h of rehydration was a Nin-like transcription factor (unigene20858_TBA), which is recognized as a central role in nitrate signalling in *A. thaliana* [27]. Based upon TF family classification and DEG screening, the ERF is the most abundant TF in family in *S. caninervis* [18], *P. patens* [15, 24] and *B. argenteum*. Among all the characterized TF families, the *B. argenteum* ERF family contained the largest number of DEGs. 17 ERFs were up-regulated 2 h after rehydration and 23 ERFs were up-regulated 24 h after rehydration. Among the 10 most up-regulated TFs, there are 4 ERFs and 3 GRAS transcription factors.

Studies have demonstrated that the AP2/ERF proteins have important functions in the transcriptional regulation of a variety of biological processes related to growth and development, as well as various responses to environmental stimuli [28]. The GRAS TF family was found throughout the plant kingdom, and these proteins have diverse roles in plant development, such as root and shoot development, gibberellic acid (GA) signalling and phytochrome A signal transduction [29]. The *B. argenteum* unigene9310_TBA is recognized as a DELLA protein, a subclass of the GRAS family, which may play important roles in gibberellic acid (GA) and light signalling [30], and was highly up-regulated 24 h after rehydration (‘R24’).

*T. ruralis* is a model moss for studying stress-responsive genes related to desiccation tolerance and rehydration, and a number of cDNAs/ESTs/transcripts have been isolated and characterized. We performed a BLASTX search against the *T. ruralis* protein sequences [31, 32], obtained from NCBI protein database, using our *B. argenteum* TACs. A total of 236 *B. argenteum* TACs were identified homologous (E-value ≤ 1e-5 and identity ≥ 50 %) with the previously characterized *T. ruralis* transcripts, including *Tr155* [33] and *Tr288* [34], *Elipa* and *Elipb* [35], *ALDH7B6* [36], *ALDH21A1* [37] and *TrDr3* [38]. For instance, a *Tr155* homolog detected in *B. argenteum* (unigene32501_TBA) was up-regulated upon rehydration, while the *TrDr3* homologs (CL2407_TBA) were down-regulated upon rehydration. A full list of the *T. ruralis* homologs detected in *B. argenteum* and their RPKM values were listed in Additional file 6.

### GO network analysis of DEGs upon rehydration

To better understand the molecular mechanisms of rehydration [1, 4, 5, 31, 32, 35, 36] and elucidate the *B. argenteum* rehydration process at the level of transcriptomics, DEG comparisons between the desiccated and rehydrated samples were performed. GO functional enrichment analysis for all DEGs from R2 (‘R2 v Dry’) and R24 (‘R24 v Dry’) were analyzed using BiNGO, a Cytoscape plugin assessing overrepresentation of ontologies in biological networks [39], using the list of all TACs from the reference transcriptome as background.

**Fig. 5:**
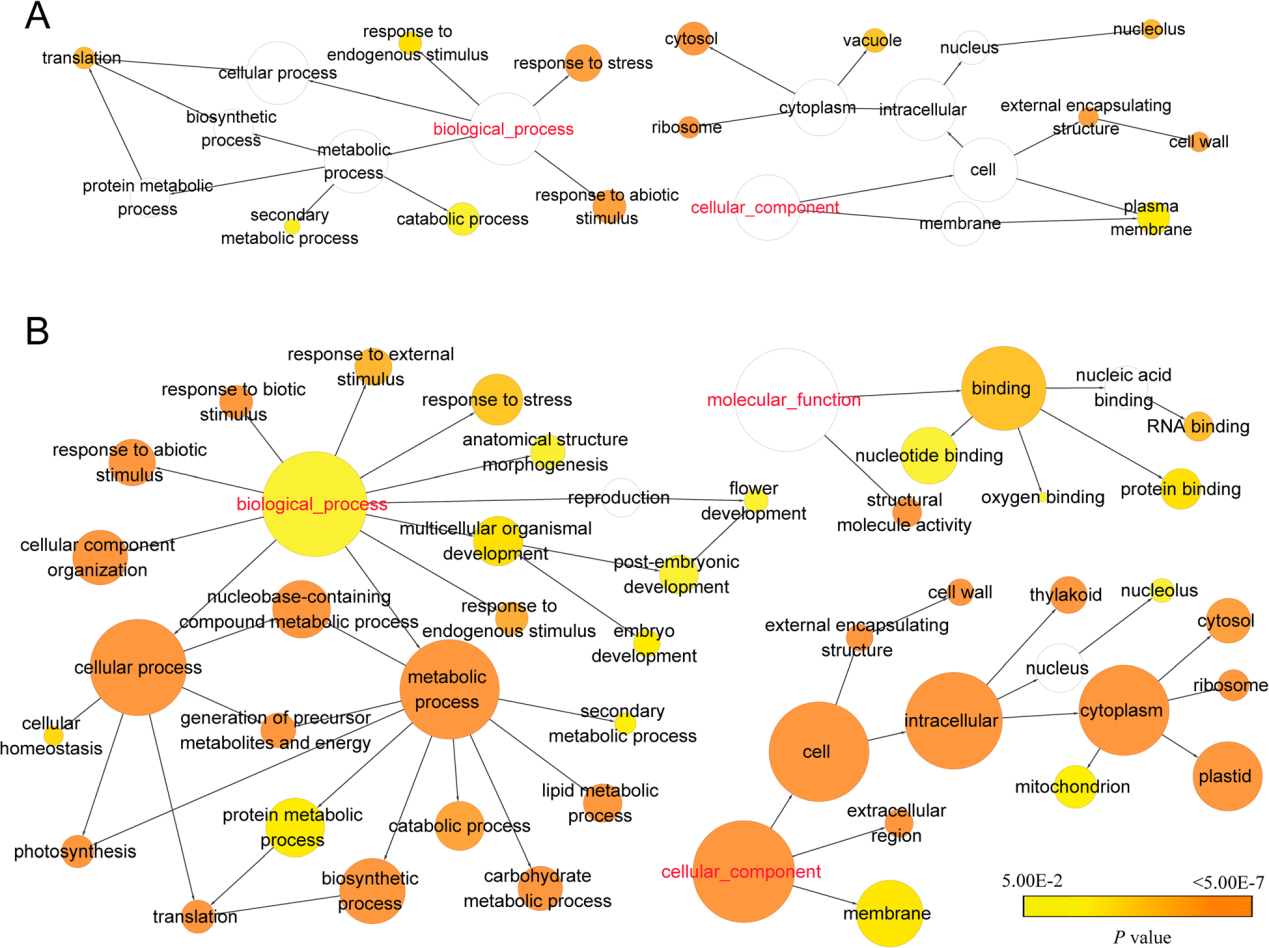
Gene Ontology network of over-representative GO-slim plant terms of rehydration up-regulated DEGs in R2 (**a**) and R24 (**b**). Node size represented gene number in node and colour of node represented p-value. White nodes were not statistically significant over-representative GO terms

In order to clearly depict the GO enrichment network, we selected the collapsed GO-slim plant terms as namespace [40]. The collapsed GO enrichment network of the up-regulated TACs in R2 and R24 is shown in Fig. 5 and Additional file 1: Table S2, and the full GO term enrichment statistical results are shown in Additional file 7. For up-regulated TACs in R2, most of the GO terms were related to stress response, such as “response to stress”, “response to abiotic stimulus” and “response to endogenous stimulus”, which were also over-represented (corrected P-value ≤ 0.05) in R24 (Fig. 5). The GO terms “vacuole” and “plasma membrane” were significantly enriched in R2 (P-values of 7.56e-5 and 3.91e-3, respectively). In contrast to R2, the most over-represented up-regulated R24 GO categories were “plastid”, “thylakoid” and “photosynthesis” (P-values were 0, 3.24e-76 and 4.45e-60, respectively). These results are consistent with the idea that alterations to membrane function and integrity is a short-term response (impacted by the in-rush of water) and that the recovery of photosynthesis is a long-term response. In both GO enrichment networks (i.e. R2 and R24), ‘ribosome’, ‘translation’ “cell wall”, “cytosol” and “nucleolus” are over-representative GO terms. These results support the idea that translation plays a central role in desiccation tolerance [41, 42].

Following rehydration, the number of up-regulated DEGs was much larger than the number of down-regulated DEGs, indicating the rehydration process was associated with accumulation of transcript rather than the non-accumulation of transcript (Fig. 4d-f). Due to the small number of down-regulated DEGs, we applied the full GO terms within the BiNGO namespace to perform GO functional enrichment analysis (Additional file 7). From the enrichment analysis, we identified a smaller number of biologically informative GO terms as compared to the up-regulated DEGs. For down-regulated DEGs in R2, the GO terms of “transmembrane transport”, “water transmembrane transporter activity”, and “water channel activity” revealed to be the most significantly over-representative terms (P-values were 3.58E-04, 4.86E-04 and 4.86E-04, respectively). Transcripts related to water transmembrane transport were less abundant upon rehydration. Concomitantly, transcripts related to ‘response to water deprivation’ and ‘response to desiccation’ were significantly down regulated 24 h after rehydration.

### Photosynthesis was significantly up-regulated upon full rehydration

**Fig. 6:**
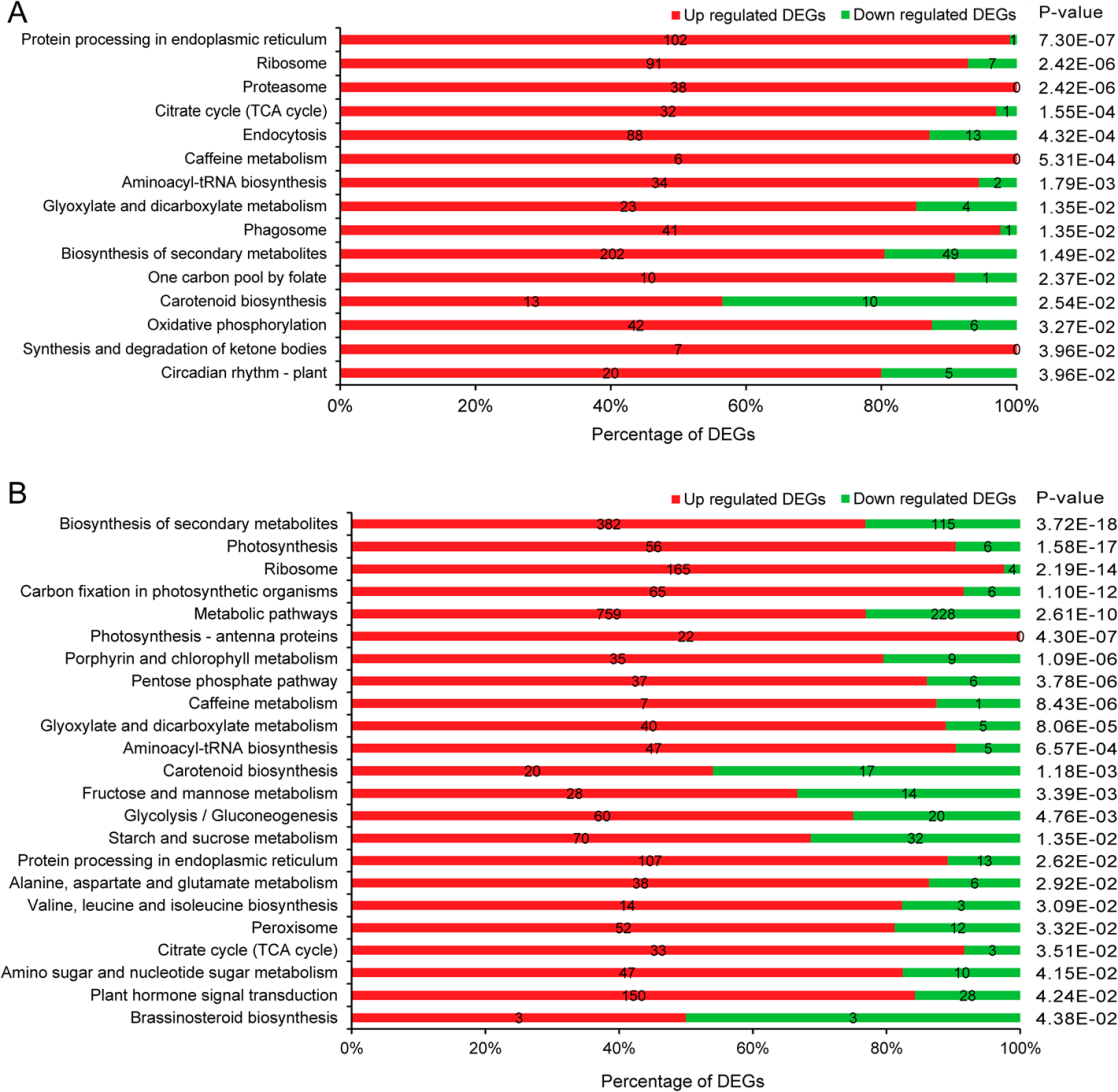
Specific significantly enriched KEGG pathways for DEGs from R2 (**a**) and R24 (**b**). Number of up- and down-regulated DEGs was shown on the bar plot. The p-values shown for each pathway were FDR corrected

To identify the metabolic pathways that were significantly altered by rehydration, we used both KEGG pathway network and MapMan to uncover the altered metabolic pathways. Significantly altered KEGG pathways were identified using a P-value based on hypergeometric distribution. As a result, 15 and 23 metabolic pathways were identified to be significantly altered after 2 h and 24 h of rehydration, respectively (Fig. 6). KEGG pathway network analysis showed that “protein processing in ER”, “ribosome”, “proteasome” and “TCA cycle” were DEG-enriched in *B. argenteum* 2 h after of rehydration. Interestingly, the TCA cycle was only found to be enhanced within 2 h upon rehydration, suggesting the necessity of mitochondrial function in plant resuscitation.

**Fig. 7:**
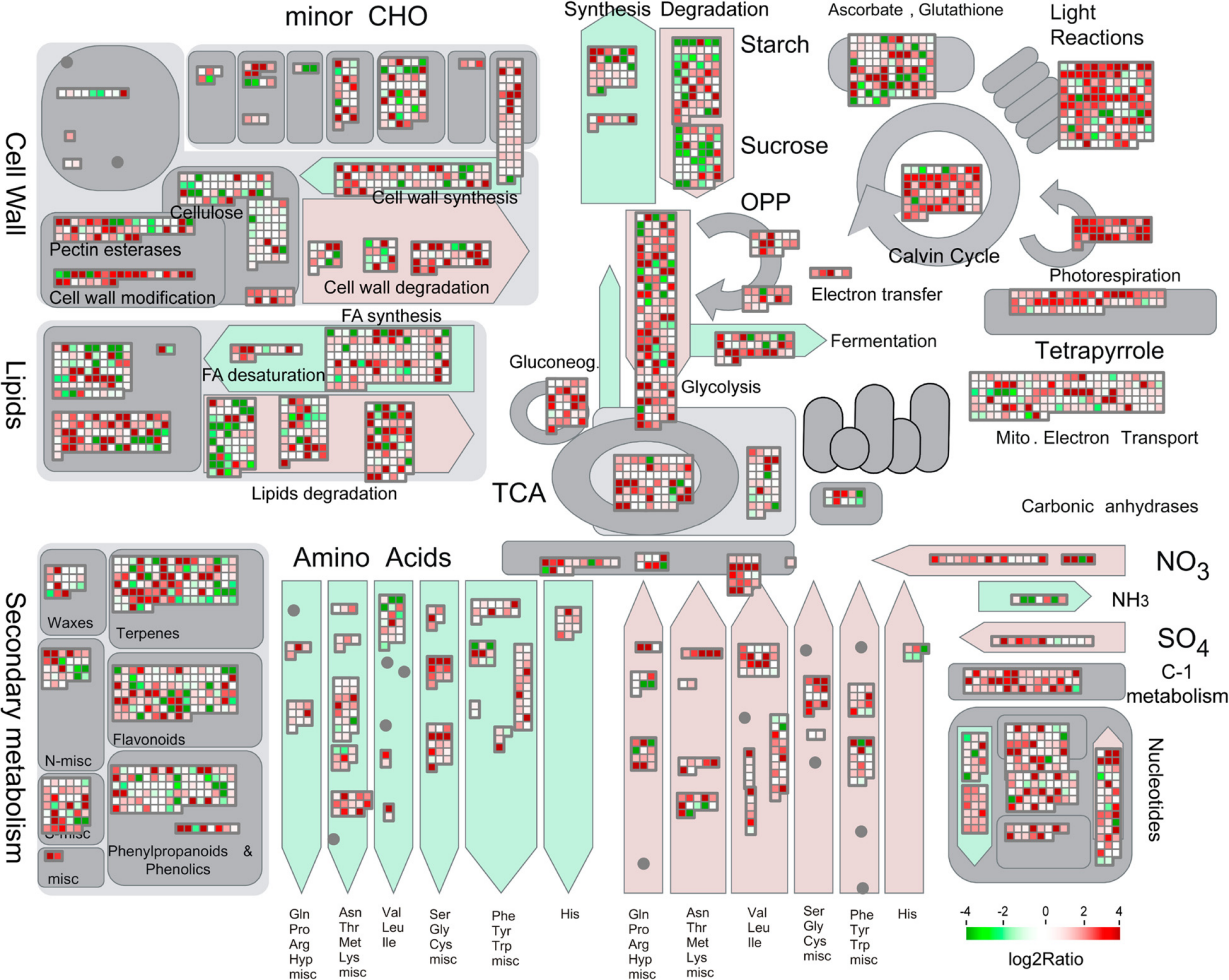
MapMan overview of *B. argenteum* cellular metabolism changes upon rehydration (R24 v Dry). Individual TACs are represented by coloured squares. The colour code scale is based on the log2 of the fold change values of each TAC. Green squares represent the down-regulated TACs, while the reds are up-regulated TACs. MapMan comparison between Dry and R2, between R2 and R24 were illustrated in Additional file 1

The metabolic pathways “photosynthesis”, “photosynthesis - antenna proteins” and “ribosome” were DEG-enriched 24 h after rehydration. DEGs anchored on the pathways of photosynthesis, antenna proteins and ribosome are depicted in Additional file 1: Figure S5 - S7. All identified differentially expressed antenna proteins (Light Harvesting Complex, LHCs), including Lhca1, Lhca2, Lhca3 and Lhca5 in photosystem I and Lhcb1 - 6 in photosystem II were induced after 24 h of rehydration (Additional file 1: Figure S6). The majority of DEGs participating in photosynthesis and ribosome/translation (Additional file 1: Figure S7) were up-regulated TACs, which is consistent with the GO enrichment network. So we could conclude the importance of translation from both GO and KEGG network enrichment analysis. Furthermore, we used MapMan to illustrate the altered expression of TACs for a number of metabolic pathways. In a MapMan comparison between R24 and Dry, TACs participating in “light reactions”, “photorespiration” were up-regulated following 24 h of rehydration (Fig. 7). MapMan comparisons between Dry and R2, R2 and R24 were depicted in Additional file 1: Figure S8 and S9.

We were able to identify and quantify the expression of a number of *B. argenteum* transcripts related to photosynthesis. PsbS (unigene32903_TBA), Lhcb3 (unigene18463_TBA, unigene18462_TBA, unigene11855_TBA), Lhc5 (CL4560 _TBA, unigene11018_TBA, unigene11017_TBA) and Lhcb6 (unigene29917_TBA) were up-regulated 24 h after rehydration. Lhc proteins are fundamental to light-harvesting and PsbS (a Lhc-like protein) is thought to play a key role in NPQ [43]. Lhca4 (unigene40215_TBA) was identified in *B. argenteum* but was not differentially expressed. In *P. patens*, Lhc4 has not been identified and Lhc5 does not accumulate under standard growth conditions [44]. The presence of the Lhcb3 and Lhcb6 protein families are limited to land plants and are postulated to play a role in the adaptation to aerial environments [45]. Previous research in *T. ruralis* has demonstrated that the Elip transcripts accumulate in response to desiccation, salinity and high light, and are down-regulated upon rehydration [35]. Elips are postulated to scavenge free Chl *a* and protect PSII from photo-oxidative damage. The gene expression profile for *B. argenteum* Elips is depicted in Additional file 1: Figure S10. Consistent with *T. ruralis*, Elip genes are associated with desiccated tissue and are down-regulated 24 h after rehydration.

### Quantitative real-time PCR validation

**Fig. 8:**
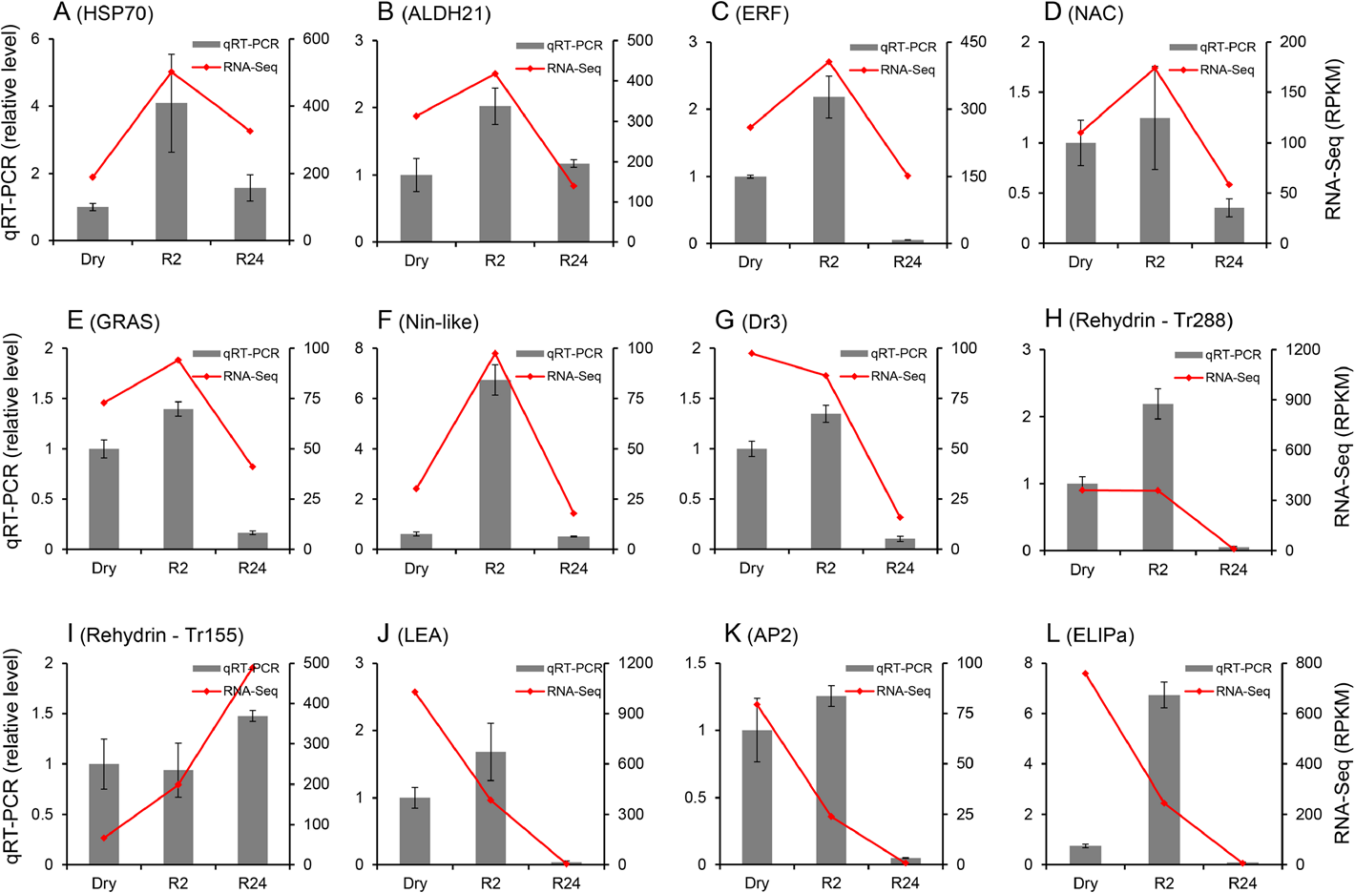
qRT-PCR validation of selected *B. argenteum* TACs. Quantitative RT-PCR analysis of selected genes. Expression level of 12 genes were quantified using RT-qPCR, including HSP70 (**a**), aldehyde dehydrogenase 21 (**b**), ERF (**c**), NAC (**d**), GRAS (**e**) and Nin-like (**f**) transcription factors, homolog of *T. ruralis* desiccation-stress related gene (TrDr3) (**g**), 2 *T. ruralis* rehydrin homologs (Tr288 (**h**) and Tr155 (**i**)), LEA (**j**), AP2 transcription factor (**k**) and Early Light Induced Protein (**l**). Quantitative gene expression data are shown as the mean ± SD. The actin and tubulin genes were used as reference genes

Quantitative RT-PCR (qPCR) analysis was performed to test the reliability of the data generated using the next generation sequencing platform. We selected 12 representative TACs based upon their hypothesized role(s) in desiccation tolerance. We validated 5 transcription factors (i.e. ERF, NAC, GRAS, Nin-like, AP2) and the *T. ruralis* homologues Tr155 [33, 41] and Tr288 [34, 41], TrDr3 [38], ELIPa [35], ALDH21 [37], Hsp70 and Lea (Fig. 8). qPCR analysis of the transcripts confirmed the DGE expression data. In 6 of the transcripts (Hsp70, Aldh21, ERF, NAC, GRAS, Nin-like), including 4 of the 5 transcription factors, expression was elevated 2 h after hydration (relative to Dry) and declined 24 h after hydration. In 4 of the transcripts (AP2, Lea, Tr288 and TrDr3) expression was elevated at both Dry and R2 declined 24 h after rehydration. The rehydrin Tr155 was the only transcript with elevated expression 24 h after rehydration. In 5 of the 36 individual analyses, notable differences in the predicted versus validated expression amounts were observed: Dry Dr3 (Fig. 8g), Dry Tr155 (Fig. 8i), Dry LEA (Fig. 8j), Dry ELIPa (Fig. 8l) and R2 Tr288 (Fig. 8h). The rehydrin Tr155 and Tr288 were both first characterized in *T. ruralis*, Tr155 was postulated to be involved in antioxidant production during rehydration [33], and Tr288 possess a "K" segment common to dehydrins (a kind of LEA protein), which accumulated upon drying and declined over time following rehydration [34]. In our qPCR analysis of the two rehydrin homologs, Tr288 was detectable at R24 and Tr155 was slightly up-regulated after rehydration.

## Conclusions

In this study we present a high-quality reference transcriptome for the desiccation-tolerant moss *B. argenteum* using next-generation, RNA-Seq technology. *De novo* transcriptome assembly generated 57,247 transcript assembly contigs (TACs). GO annotation of the *B. argenteum* transcriptome indicates the enhancement of sequence abundance related to ‘envelope’, ‘biological regulation’ and ‘response to stimulus’. GO functional and pathway enrichment analysis of the DEGs demonstrated the induction of genes related to stress response and support the hypothesis that the rapid recovery of photosynthesis following rehydration, and *de novo* protein synthesis plays an important role in the recovery of *B. argenteum* following rehydration. The data extends our knowledge of bryophyte transcriptomes and provides an insight into the molecular aspects of the rehydration process.

## Materials and methods

### Plant material, culture and drying treatments

*Bryum argenteum* Hedw. gametophores were collected from the Gurbantunggut Desert of Xinjiang Uygur Autonomous Region of China (44°32′30″N, 88°6′42″E) and harvested and stored as described previously for *Syntrichia caninervis* gametophores [18]. Voucher specimens are maintained in the Department of Plant Biology, Southern Illinois University (Carbondale, IL, USA). *B. argenteum* gametophyte tissue was cultured on solid Knop media (0.25 g/L KH2PO4, KCl, MgSO4·7H2O, 1 g/L Ca(NO3)2·4H2O and 0.0125 g/L FeSO4·7H2O, pH adjusted to 5.8) in 9 cm Petri dishes. 60 explants were cultured at 25 °C with a 16 h photoperiod (under cool white fluorescent light, ~ 4000 lux). Gametophytes of *B. argenteum* were prepared for experimentation as described [18]. Desiccated moss tissue was obtained by placing gametophytes on dry filter paper in an open Petri dish exposed to 20 % RH for 24 h. This drying regime could result in the attainment of the gametophytes in desiccated state (Dry) that losses > 95 % of its wet weight. Then the desiccated gametophytes (Dry) were rehydrated for 2 h (R2) with deionized water at 18 °C in the light. Samples were also taken for the fully rehydrated for 24 h (R24) gametophytes.

### RNA extraction, cDNA library construction and RNA sequencing

For RNA sequencing, total RNAs were extracted using TRIzol reagent (Invitrogen, Carlsbad, CA, USA). The quality of total RNAs were checked using the NanoDrop 2000 Spectrometer and Agilent 2100 Bioanalyzer. High quality RNA samples were sent to Beijing Genomics Institute (BGI, Shenzhen, China) for cDNA libraries construction and sequencing using Illumina HiSeq™ 2000. For the purpose of this study, a two-step approach was applied to analyze the *B. argenteum* rehydration process. First, an integrated reference transcriptome was reconstructed and used for gene function annotation. The cDNA library for transcriptome generation was prepared by pooling the *B. argenteum* gametophores from different rehydration stages (Dry, R2 and R24). Second, we constructed 3 digital gene expression (DGE) libraries from different rehydration stages, including desiccated (Dry), rehydrated for 2 h (R2) and rehydrated for 24 h (R24) separately. The cDNA library construction method and Illumina deep-sequencing processes were the same as described previously [18, 45]. All the sequencing reads of the pooled transcriptome and the 3 DGE libraries have been deposited at the NCBI Sequence Read Archive (SRA) repository with accessions of SRR1763242, SRR1763243, SRR1763244 and SRR1763245.

### *De novo* transcriptome assembly and annotation

Raw reads of low quality were eliminated or trimmed to generate clean reads, which were used for *de novo* transcriptome assembly using Trinity software [46], with optimized single k-mer length of 25. Then, the contigs generated by Trinity were further clustered with TGICL software [47] to get sequences (final transcript assembly contigs, TACs) that cannot be extended on either end, with parameters of “-l 40 -c 10 -v 20”. Final reconstructed TACs with length equal to or longer than 200 bp were retained for further analysis.

For annotating the *B. argenteum* transcriptome, TACs of the final assembly were searched against NCBI-nr, Swiss-prot, KEGG and COG databases using BLASTX with an E-value cut-off of 1e-5. Then all the TACs were divided into two groups: ‘with BLAST’ and ‘without BLAST’. The coding region for each TAC and polypeptide sequences were then obtained using the method as described previously [18]. To evaluate the protein coding potential of the two groups of TACs, we used the program CPAT (Coding Potential Assessment Tool) v 1.2 [19], which calculates the coding potential for reconstructed transcripts. Furthermore, ORF size, GC content and expression levels were compared between the two sets of TACs. According to the NCBI-nr annotation results, the top BLASTX hits were used to identify putative homologous proteins and annotate each TAC with gene ontology (GO) terms using Blast2GO [48] with default parameters. Then the web-based program WEGO [49] was used to perform GO functional classification to illustrate the distribution of gene functional categories, according to molecular function, biological process, and cellular component ontologies.

To assess the completeness of the transcriptome assembly, all TACs were searched against the list of 357 *Arabidopsis thaliana* proteins that are conserved as single copy genes across all eukaryotes (i.e. ultra-conserved orthologs, UCOS, http://compgenomics.ucdavis.edu/compositae_reference.php), using BLASTX (E-value ≤ 1e-6, and identity ≥30 %). To investigate metabolic pathway annotation, we performed a BLASTX of TACs against the KEGG database [22]; the resulting KEGG orthology and EC accession numbers were used to colour and retrieve the corresponding KEGG pathway maps.

For Pfam domain/family annotation, the predicted protein sequences were submitted to search against HMM profiles contained in the Pfam database (version 27.0) [23] using the software package HMMER v3.0 [50, 51]. To resolve complex overlapping protein domains, only the most significant (lowest E-value) match within the clan was reported. The *B. argenteum* transcription factors were predicted using PlantTFDB v3.0 [24]. The putative transcription factors of *B. argenteum* were initially identified, including proteins that contain a DNA binding domain (inferred from Pfam annotation) or gave a positive BLASTP hit (E-value ≤ 1e-5) with recorded *P. patens* or *A. thaliana* transcription factors. Deduced polypeptide sequences were then submitted to the PlantTFDB prediction server (http://planttfdb.cbi.pku.edu.cn/prediction.php) for further classification and validation.

### Screening and analysis of DEGs

All reconstructed TACs were used as the reference transcriptome sequences for the evaluation of the expression values. The 49 bp reads sequenced from three individual DGE (Dry, R2 and R24) were mapped against the reference transcriptome with SOAP (v2.21), a short read aligner [52], with specific parameters of: “−m 0 -x 500 -s 40 -l 35 -v 5 -r 2”. Mismatches of no more than two bases were allowed in the alignment. Expression level of *B. argenteum* TACs was normalized using RPKM method [25]. Rehydration regulated DEGs were identified with FDR < 0.001 and absolute value of the log2 Fold-Change ≥ 1 [26, 53].

The GO enrichment was analyzed with BiNGO plugin [39] in Cytoscape [54], using hypergeometric test for statistical analysis. For P-value correction, we selected the FDR correction method. GO terms with corrected p-value ≤ 0.05 were considered significantly over-representative and shown as colored nodes in the enrichment network. And significantly altered KEGG pathway were identified using a P-value based on hypergeometric distribution. All metabolic pathways with a FDR corrected P-value smaller than 0.05 were reported as significantly altered upon rehydration.

Furthermore, scrutiny of TAC expression changes was performed with MapMan [55, 56]. The deduced polypeptide sequences were submitted to Mercator webserver [57] to classify them into MapMan functional plant categories. For color-coded representation (heat map) in MapMan, the log2 transformed of the fold-change for each TAC was employed. Deduced polypeptide sequences shorter than 50 amino acids were excluded to generate the MapMan metabolic pathway maps.

### Quantitative real-time PCR (qRT-PCR) analysis

For quantitative real-time PCR, cDNA was synthesized using the PrimeScript™ RT reagent Kit (TaKaRa, China) according to the manufacturer's protocol with random hexamer primers. PCR primers were designed using the Primer Premier v5.0 software (Premier Biosoft, USA). The specificity of primer pairs were confirmed by BLASTN with non-redundant unigene set of *B. argenteum* transcripts, and melting curve analysis was performed for each primer pair before further analyses. qRT-PCR was performed using the QuantiFast SYBR Green PCR Kit (Qiagen, Germany) using a 20 μl reaction volume following the manufacturer's protocol, fluorescence intensity was measured in the CFX96™ Real-Time System (Bio-Rad, USA). Triplicates of each reaction were performed. The target gene expression levels were normalized using the actin and α-tubulin genes as internal references. The relative abundance of transcript levels was calculated relative to the reference genes according to the 2^-ΔΔCt^ method [58]. All data are shown as the mean ± SD and all PCR primer information is provided in Additional file 1: Table S3.

## Additional files

Additional file 1:
**Figure S1** **-** **S10 and TableS1** **-** **S3 mentioned in the article.** **Figure S1** **.** Distribution of Ortholog Hit Ratios, calculated on the *B. argenteum* TACs with an positive BLASTX hit with *P. patens*. **Figure S2.** Venn diagram showing the BLASTX results of the *B. argenteum* TACs against four protein databases. **Figure S3.** Species distribution of the top BLASTX hits obtained using the *B. argenteum* transcriptome. **Figure S4.** Functional classifications of GO terms of *B. argenteum* TACs detected from Dry, R2 and R24. **Figure S5.** Differentially expressed *B. argenteum* TACs after 24 h rehydration of the photosynthesis metabolic pathway by KEGG annotation. **Figure S6.** Differentially expressed *B. argenteum* TACs after 24 h of rehydration of the photosynthesis – antenna proteins by KEGG annotation. **Figure S7.** Differentially expressed *B. argenteum* TACs after 24 h of rehydration of the ribosome proteins by KEGG annotation. **Figure S8.** MapMan overview of *B. argenteum* cellular metabolism changes following rehydrating 2 h (R2 v Dry). **Figure S9**. MapMan overview of *B. argenteum* cellular metabolism changes between two rehydrated samples (R24 v R2). **Figure S10.** Hierarchical cluster analyses of the putative early light induced proteins following rehydration. **Table S1.** COG functional classification of *B. argenteum* TACs. **Table S2.** Statistically over-representative GO-slim terms of rehydration up-regulated genes in R2 and R24. **Table S3.** Primers used for quantitative real-time PCR (qRT-PCR) analysis. Additional file 2:
**Complete list of** ***B. argenteum*** **TACs with BLASTX hits.** Including BLASTX search hits in NCBI-Nr, Swiss-Prot, KEGG and COG databases. Additional file 3:
**Pfam domain/family annotation of** ***B. argenteum*** **TACs and statistics of** ***B. argenteum*** **TACs per family (2 sheets).**
Additional file 4:
**All** ***B. argenteum*** **transcription factors annotated using PlantTFDB and the lists of differentially expressed TFs (2 sheets).**
Additional file 5:
**Digital gene expression profiles for Dry, R2 and R24 and the full list of differentially expressed genes by pairwise comparisons (4 sheets).**
Additional file 6:
**Homologs of** ***T. ruralis*** **transcripts detected in** ***B. argenteum*** **and their expression RPKM values during rehydration.**
Additional file 7:
**Significantly** **over-representative** **GO terms for** **up-** **and** **down-regulated** **genes.** Hypergeometric test was performed for the DEGs to identify the over-representative GO terms using BiNGO. All the GO terms with a FDR corrected P-value less than 0.05 were shown in the tables (2 sheets).

## Abbreviations

BSC: Biological soil crust
DEG: Differentially expressed genes
DGE: Digital gene expression
GO: Gene ontology
RPKM: Reads per kilobase per million mapped reads
TAC: Transcript assembly contig
TF: Transcription factor
