# Supplementary material for: De novo transcriptome characterization and gene expression profiling of the desiccation tolerant moss Bryum argenteum following rehydration
Source: BMC Genomics. 2015 May 28;16(1):416. doi: 10.1186/s12864-015-1633-y (PMC4445806; doi:10.1186/s12864-015-1633-y)
Supplement: Additional file 1: — Figure S1 - S10 and TableS1 - S3 mentioned in the article. Figure S1 . Distribution of Ortholog Hit Ratios, calculated on the B. argenteum TACs with an positive BLASTX hit with P. patens. Figure S2. Venn diagram showing the BLASTX results of the B. argenteum TACs against four protein databases. Figure S3. Species distribution of the top BLASTX hits obtained using the B. argenteum transcriptome. Figure S4. Functional classifications of GO terms of B. argenteum TACs detected from Dry, R2 and R24. Figure S5. Differentially expressed B. argenteum TACs after 24 h rehydration of the photosynthesis metabolic pathway by KEGG annotation. Figure S6. Differentially expressed B. argenteum TACs after 24 h of rehydration of the photosynthesis – antenna proteins by KEGG annotation. Figure S7. Differentially expressed B. argenteum TACs after 24 h of rehydration of the ribosome proteins by KEGG annotation. Figure S8. MapMan overview of B. argenteum cellular metabolism changes following rehydrating 2 h (R2 v Dry). Figure S9. MapMan overview of B. argenteum cellular metabolism changes between two rehydrated samples (R24 v R2). Figure S10. Hierarchical cluster analyses of the putative early light induced proteins following rehydration. Table S1. COG functional classification of B. argenteum TACs. Table S2. Statistically over-representative GO-slim terms of rehydration up-regulated genes in R2 and R24. Table S3. Primers used for quantitative real-time PCR (qRT-PCR) analysis. [file 12864_2015_1633_MOESM1_ESM.pdf]

Supplementary Information for

De novo transcriptome characterization and gene expression profiling of the desiccation tolerant moss *Bryum argenteum* following rehydration

Bei Gao, Daoyuan Zhang*, Xiaoshuang Li, Honglan Yang, Yuanming Zhang, Andrew J. Wood

Supplementary Figures and Tables.

Supplemental Figures:

Figure S1: Distribution of Ortholog Hit Ratios, calculated on the *B. argenteum* TACs with a positive BLASTX hit with *P. patens*.

Figure S2: Venn diagram showing the BLASTX results of the *B. argenteum* TACs against four protein databases.

Figure S3: Species distribution of the top BLASTX hits obtained using the *B. argenteum* transcriptome.

Figure S4: Functional classifications of GO terms of *B. argenteum* TACs detected from Dry, R2 and R24.

Figure S5: Differentially expressed *B. argenteum* TACs after 24 hours rehydration of the photosynthesis metabolic pathway by KEGG annotation.

Figure S6: Differentially expressed *B. argenteum* TACs after 24 hours of rehydration of the photosynthesis – antenna proteins by KEGG annotation.

Figure S7: Differentially expressed *B. argenteum* TACs after 24 hours of rehydration of the ribosome proteins by KEGG annotation.

Figure S8: MapMan overview of *B. argenteum* cellular metabolism changes following rehydrating 2 hours (R2 v Dry).

Figure S9: MapMan overview of B. argenteum cellular metabolism changes between two rehydrated samples(R24 v R2).

Figure S10: Hierarchical cluster analyses of the putative early light induced proteins following rehydration.

Supplemental Tables:

Table S1: COG functional classification of *B. argenteum* TACs.

Table S2: Statistically over-representative GO-slim terms of rehydration up-regulated genes in R2 and R24.

Table S3: Primers used for quantitative real-time PCR (qRT-PCR) analysis.

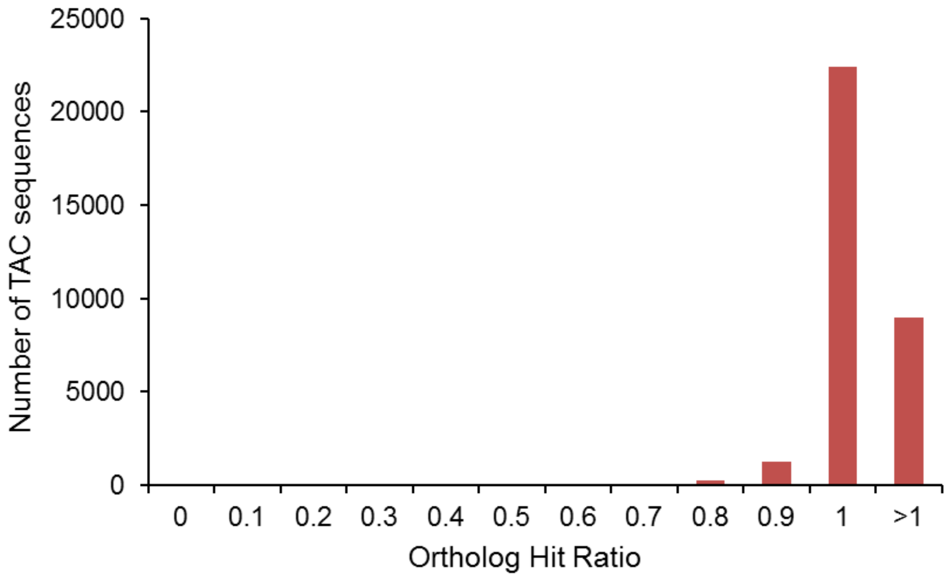

Figure S1 – Ortholog Hit Ratio, calculated on the *B. argenteum* TACs with an positive BLASTX hit with *P. patens*. The ratio of length between *B. argenteum* TACs and the *P. patens* orthologs derived from its genome is reported on the x-axis, the number of TACs observed in each ratio category is shown on the y-axis.

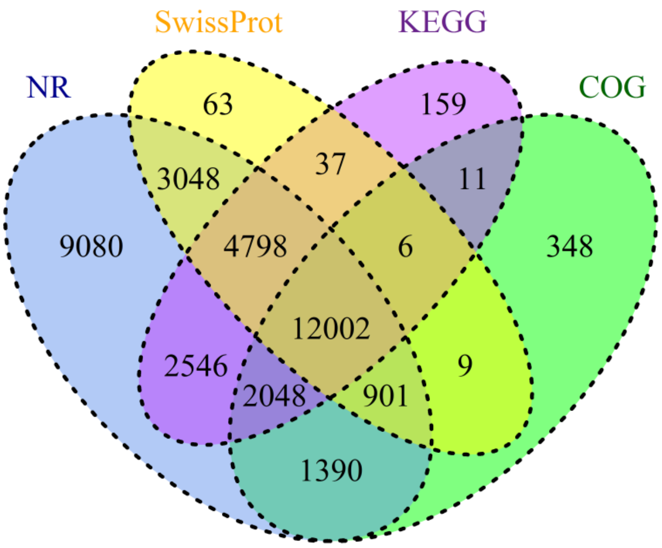

Figure S2 – Venn diagram showing the BLASTX results of the *B. argenteum* transcriptome against four protein databases.

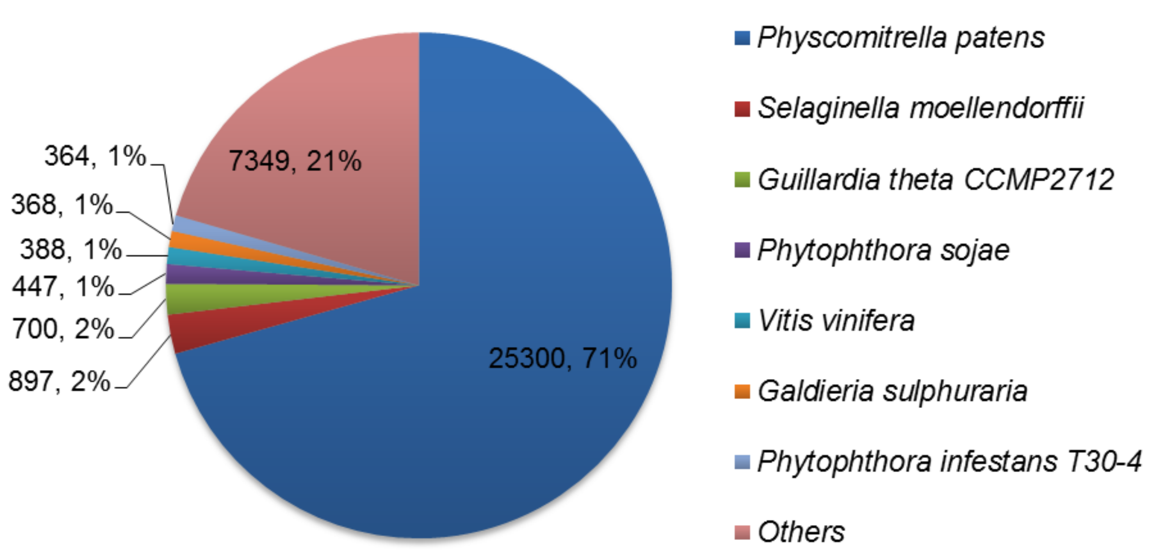

Figure S3 – Species distribution of the top BLASTX hits obtained using the *B. argenteum* transcriptome. Top scoring BLASTX hits against the NCBI-nr protein database are depicted. The number of BLAST hits per species is shown on the x-axis. The 7 most represented species with proportions of more than 1% are shown in this graph.

Table S1 - COG functional classification of the *B. argenteum* transcript assembly contigs

Functional-Categories	Code	TACs (n)
RNA processing and modification	A	205
Chromatin structure and dynamics	B	458
Energy production and conversion	C	1167
Cell cycle control, cell division, chromosome partitioning	D	1406
Amino acid transport and metabolism	E	1218
Nucleotide transport and metabolism	F	313
Carbohydrate transport and metabolism	G	1951
Coenzyme transport and metabolism	H	619
Lipid transport and metabolism	I	892
Translation, ribosomal structure and biogenesis	J	2413
Transcription	K	2678
Replication, recombination and repair	L	2540
Cell wall/membrane/envelope biogenesis	M	1518
Cell motility	N	366
Posttranslational modification, protein turnover, chaperones	O	2025
Inorganic ion transport and metabolism	P	872
Secondary metabolites biosynthesis, transport and catabolism	Q	910
General function prediction only	R	5271
Function unknown	S	1509
Signal transduction mechanisms	T	2133
Intracellular trafficking, secretion, and vesicular transport	U	1146
Defense mechanisms	V	343
Extracellular structures	W	61
Nuclear structure	Y	18
Cytoskeleton	Z	503

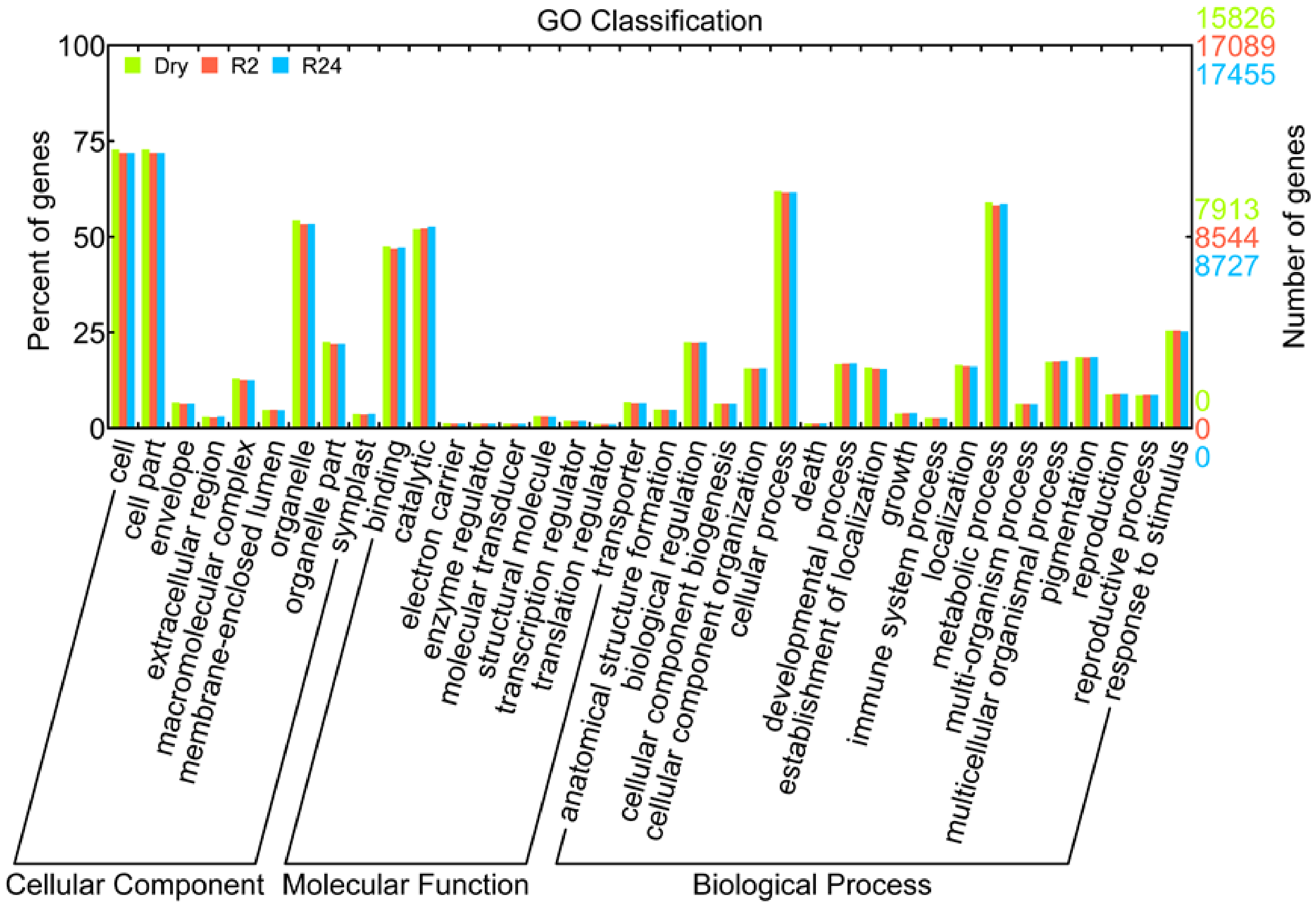

Figure S4 – Functional classifications of GO terms of *B. argenteum* TACs detected from Dry, R2 and R24.

Table S2 - Statistically over-representative GO-slim terms of rehydration up-regulated genes in R2 and R24

GO ID	Description	Total	Up-regulated DEGs in R24		Up-regulated DEGs in R24	
			Frequency	P-value*	Frequency	P-value
9536	plastid	4470/23509 19.0%	1055/3089 34.1%	0.00E+00	--	--
9579	thylakoid	628/23509 2.6%	269/3089 8.7%	3.24E-76	--	--
15979	photosynthesis	436/23509 1.8%	197/3089 6.3%	4.45E-60	--	--
5737	cytoplasm	11552/23509 49.1%	1831/3089 59.2%	1.29E-32	--	--
6091	generation of precursor metabolites and energy	835/23509 3.5%	237/3089 7.6%	2.72E-31	--	--
5840	ribosome	646/23509 2.7%	184/3089 5.9%	1.94E-24	93/1552 5.9%	7.71E-11
9058	biosynthetic process	5360/23509 22.7%	936/3089 30.3%	2.91E-24	--	--
6412	translation	875/23509 3.7%	212/3089 6.8%	1.07E-18	94/1552 6.0%	2.30E-05
5622	intracellular	14586/23509 62.0%	2132/3089 69.0%	2.60E-17	--	--
5623	cell	15753/23509 67.0%	2264/3089 73.2%	3.20E-15	--	--
5198	structural molecule activity	629/23509 2.6%	154/3089 4.9%	3.83E-14	--	--
9987	cellular process	14567/23509 61.9%	2095/3089 67.8%	1.68E-12	--	--
5975	carbohydrate metabolic process	2321/23509 9.8%	406/3089 13.1%	1.24E-09	--	--
16043	cellular component organization	3920/23509 16.6%	638/3089 20.6%	1.98E-09	--	--

6139	nucleobase-containing compound metabolic process	4459/23509 18.9%	714/3089 23.1%	2.35E-09	--	--
5618	cell wall	590/23509 2.5%	129/3089 4.1%	1.36E-08	74/1552 4.7%	1.42E-06
9607	response to biotic stimulus	1084/23509 4.6%	209/3089 6.7%	2.11E-08	--	--
30312	external encapsulating structure	596/23509 2.5%	129/3089 4.1%	2.33E-08	74/1552 4.7%	1.78E-06
5576	extracellular region	698/23509 2.9%	146/3089 4.7%	2.38E-08	--	--
8152	metabolic process	16060/23509 68.3%	2243/3089 72.6%	6.70E-08	--	--
6629	lipid metabolic process	1685/23509 7.1%	296/3089 9.5%	1.98E-07	--	--
5575	cellular_component	16989/23509 72.2%	2355/3089 76.2%	1.98E-07	--	--
9628	response to abiotic stimulus	2717/23509 11.5%	446/3089 14.4%	3.97E-07	247/1552 15.9%	1.42E-06
5829	cytosol	2530/23509 10.7%	415/3089 13.4%	1.25E-06	250/1552 16.1%	8.74E-10
9056	catabolic process	3172/23509 13.4%	505/3089 16.3%	2.32E-06	244/1552 15.7%	3.78E-02
9719	response to endogenous stimulus	1128/23509 4.7%	202/3089 6.5%	6.82E-06	106/1552 6.8%	1.29E-03
9605	response to external stimulus	1606/23509 6.8%	271/3089 8.7%	1.79E-05	--	--
3723	RNA binding	889/23509 3.7%	161/3089 5.2%	3.73E-05	--	--
5488	binding	11293/23509 48.0%	1591/3089 51.5%	6.10E-05	--	--
6950	response to stress	3624/23509 15.4%	552/3089 17.8%	1.15E-04	316/1552 20.3%	1.28E-06
19725	cellular homeostasis	296/23509 1.2%	61/3089 1.9%	6.36E-04	--	--
5515	protein binding	2111/23509 8.9%	328/3089 10.6%	1.31E-03	--	--
7275	multicellular organismal development	3435/23509 14.6%	513/3089 16.6%	1.38E-03	--	--
16020	membrane	6854/23509 29.1%	974/3089 31.5%	2.84E-03	--	--
9790	embryo development	759/23509 3.2%	128/3089 4.1%	4.33E-03	--	--
19538	protein metabolic process	5163/23509 21.9%	742/3089 24.0%	4.53E-03	--	--
19748	secondary metabolic process	416/23509 1.7%	75/3089 2.4%	6.53E-03	41/1552 2.6%	4.79E-02
5739	mitochondrion	2496/23509 10.6%	373/3089 12.0%	7.24E-03	--	--
9908	flower development	577/23509 2.4%	97/3089 3.1%	1.45E-02	--	--
8150	biological_process	20035/23509 85.2%	2677/3089 86.6%	1.81E-02	--	--
5730	nucleolus	603/23509 2.5%	100/3089 3.2%	1.81E-02	71/1552 4.5%	2.00E-05
9791	post-embryonic development	1998/23509 8.4%	298/3089 9.6%	1.87E-02	--	-
166	nucleotide binding	4825/23509 20.5%	683/3089 22.1%	2.29E-02	--	-
19825	oxygen binding	20/23509 0.0%	7/3089 0.2%	2.31E-02	--	-
9653	Anatomical structure morphogenesis	1521/23509 6.4%	228/3089 7.3%	3.34E-02	--	-
5773	vacuole	1198/23509 5.0%	--	--	118/1552 7.6%	7.56E-05
5886	plasma membrane	2853/23509 12.1%	--	--	231/1552 14.8%	3.91E-03

* The P-value shown in the table was FDR corrected in BiNGO.

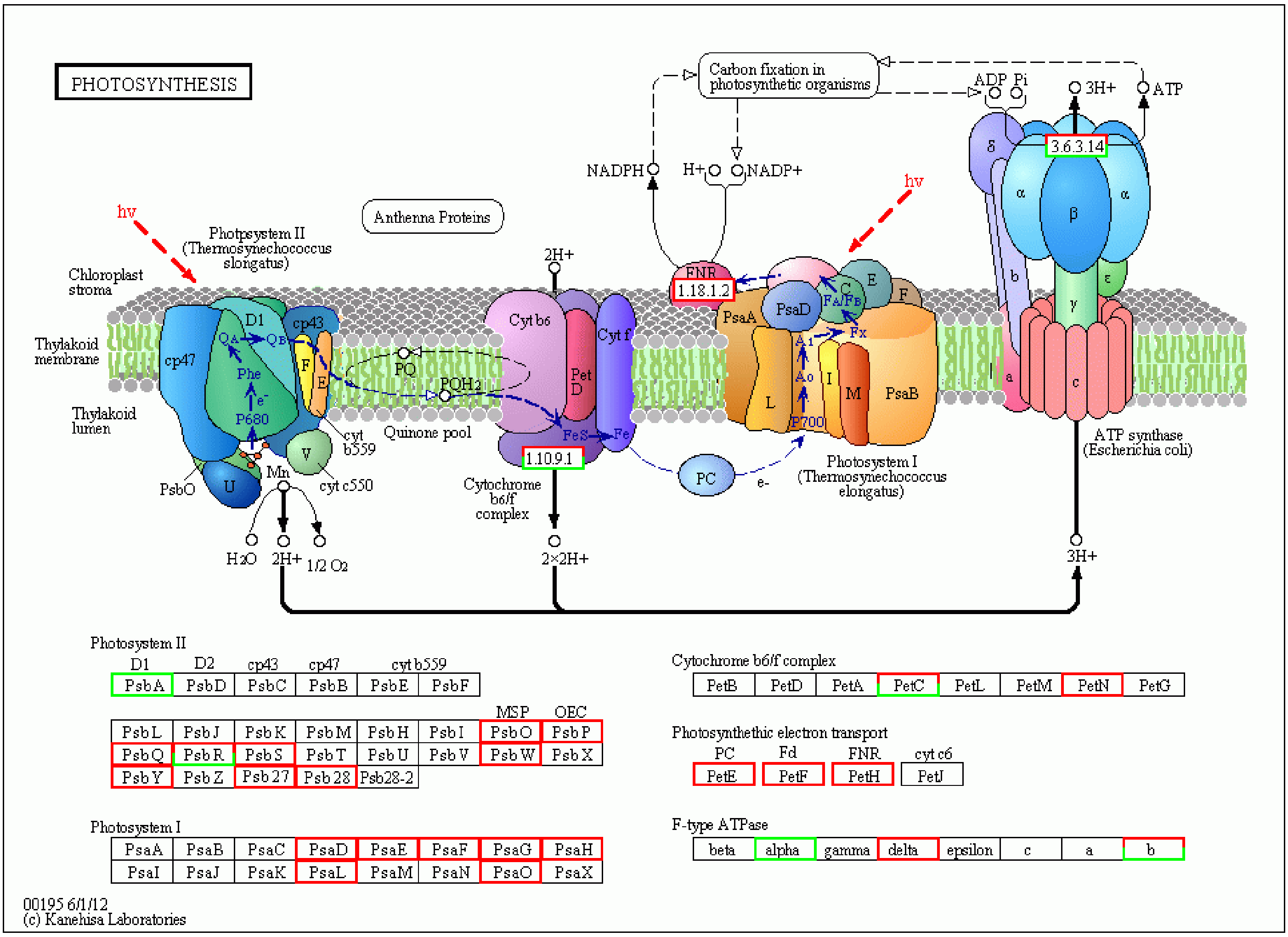

Figure S5 – Differentially expressed *B. argenteum* TACs after 24 hours rehydration of the photosynthesis metabolic pathway by KEGG annotation. Up-regulated TACs for each protein family were colored as red, and green for down-regulated TACs.

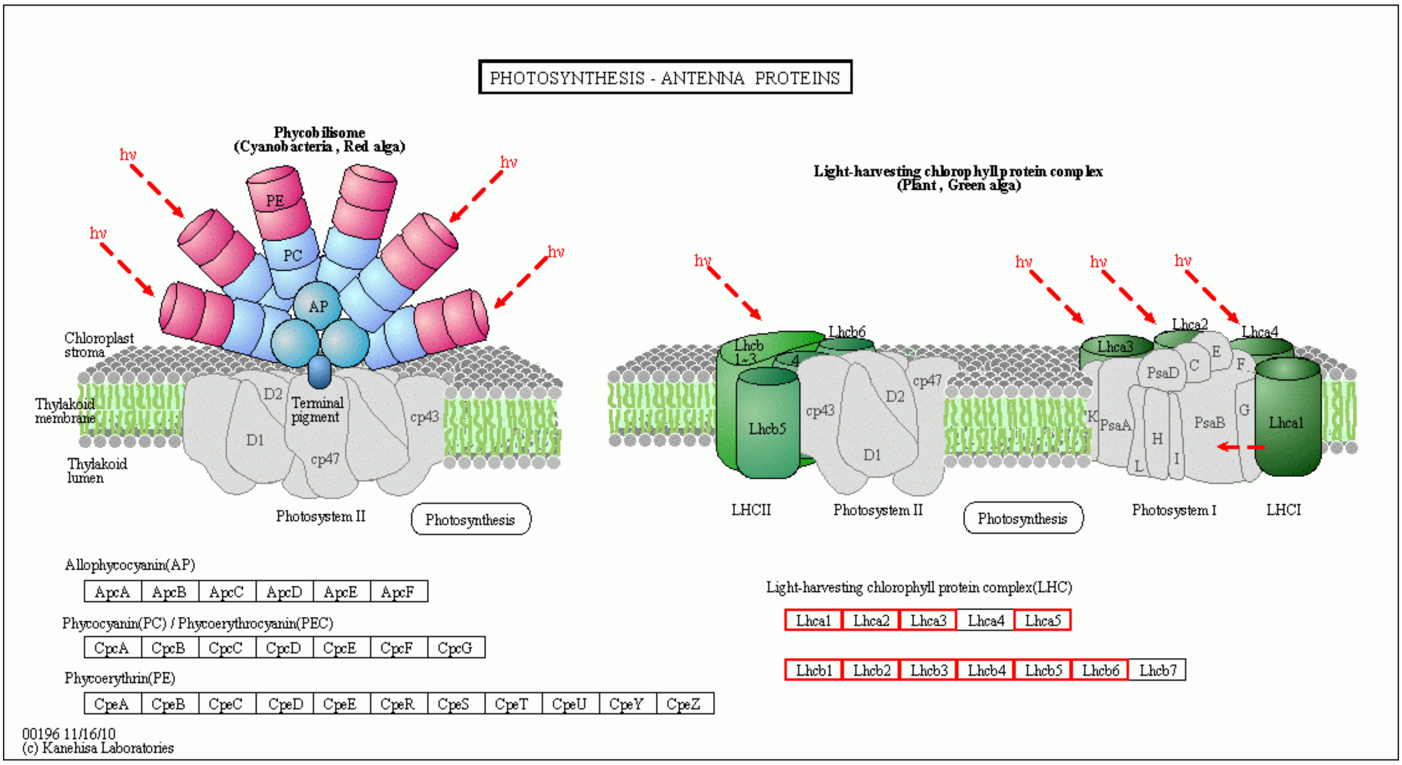

Figure S6 – Differentially expressed *B. argenteum* TACs after 24 hours of rehydration of the photosynthesis – antenna proteins by KEGG annotation. Up-regulated TACs for each protein family were colored as red, and green for down-regulated TACs.

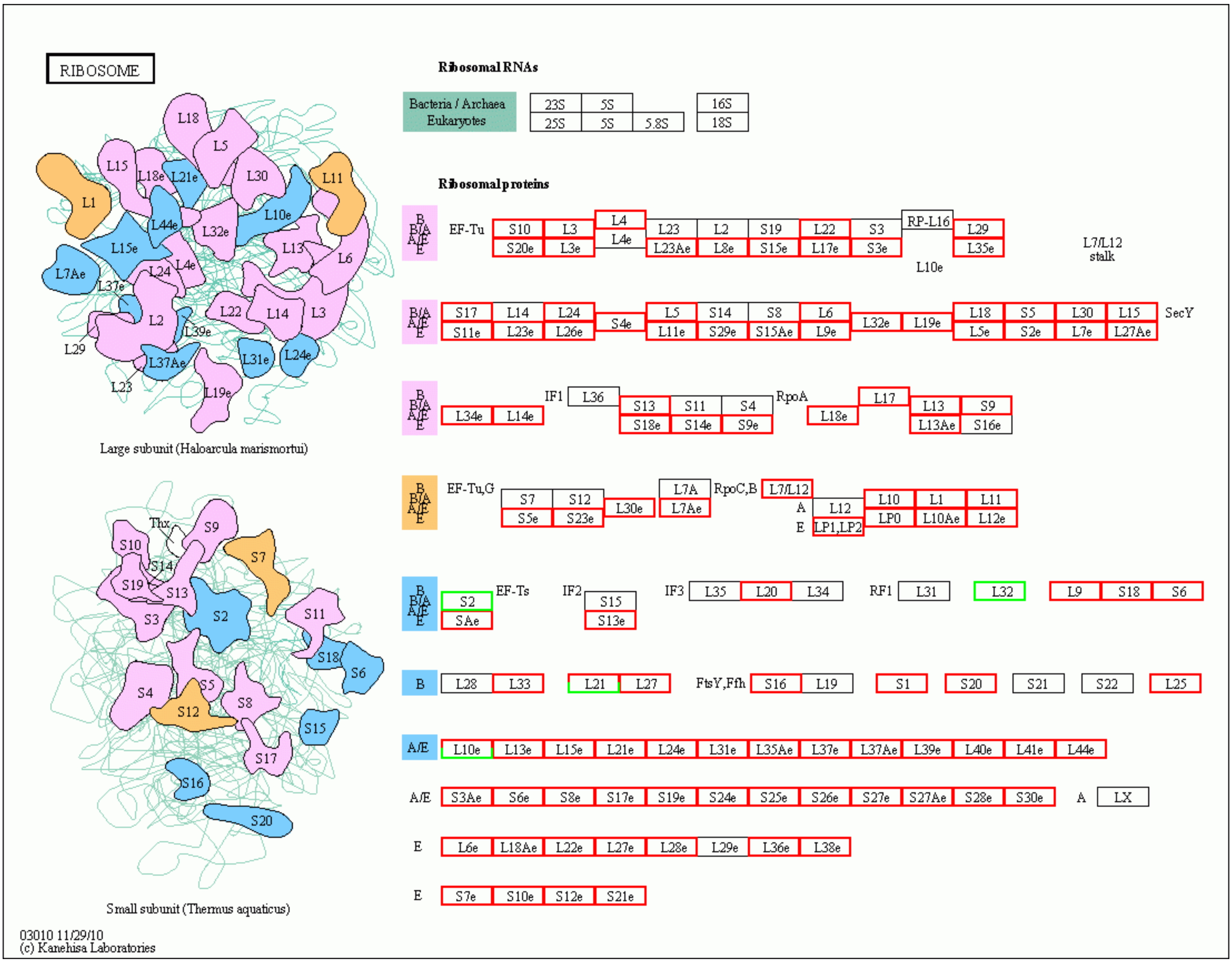

Figure S7 – Differentially expressed *B. argenteum* TACs after 24 hours of rehydration of the ribosome proteins by KEGG annotation. Up-regulated TACs for each protein family were colored as red, and green for down-regulated TACs.

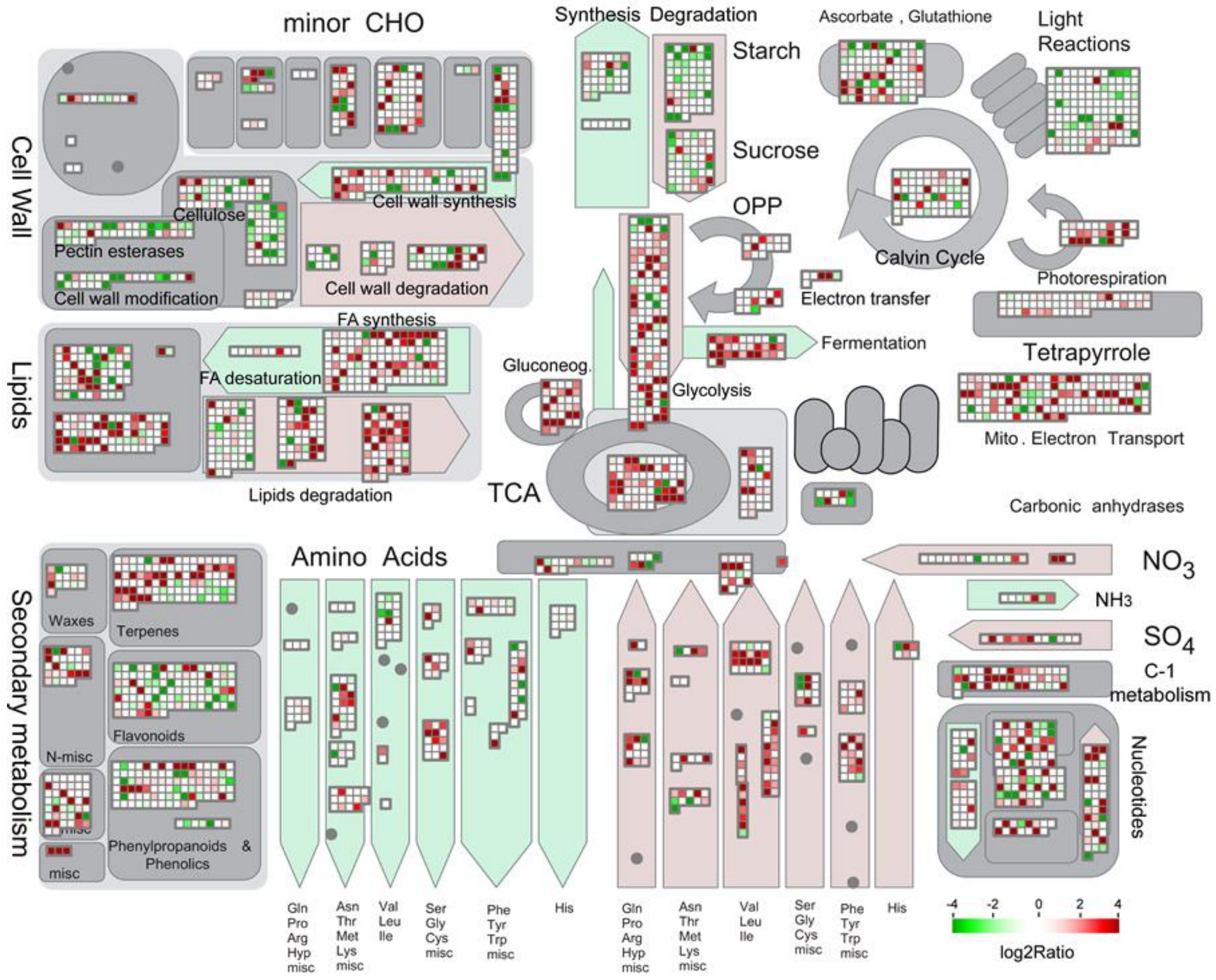

Figure S8 - MapMan overview of *B. argenteum* cellular metabolism changes following rehydrating 2 hours (R2 v Dry). Individual TACs are represented by colored squares. The color code scale is based on the log2 of the fold change values of each TAC. Gereen squares represent the down-regulated TACs, while the reds are up-regulated TACs.

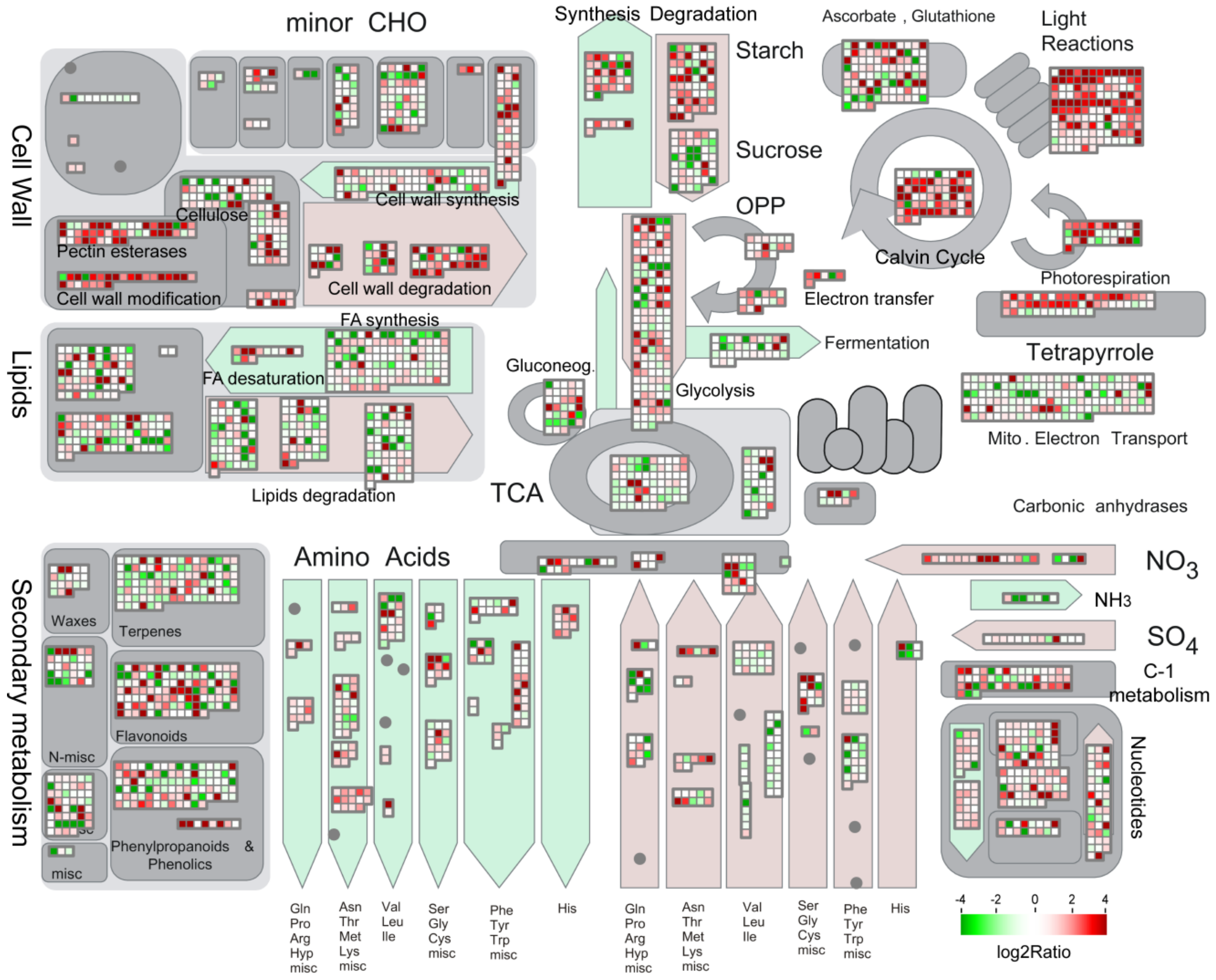

Figure S9 - MapMan overview of *B. argenteum* cellular metabolism changes between two rehydrated samples(R24 v R2). Individual TACs are represented by colored squares. The color code scale is based on the log2 of the fold change values of each TAC. Gereen squares represent the down-regulated TACs, while the reds are up-regulated TACs.

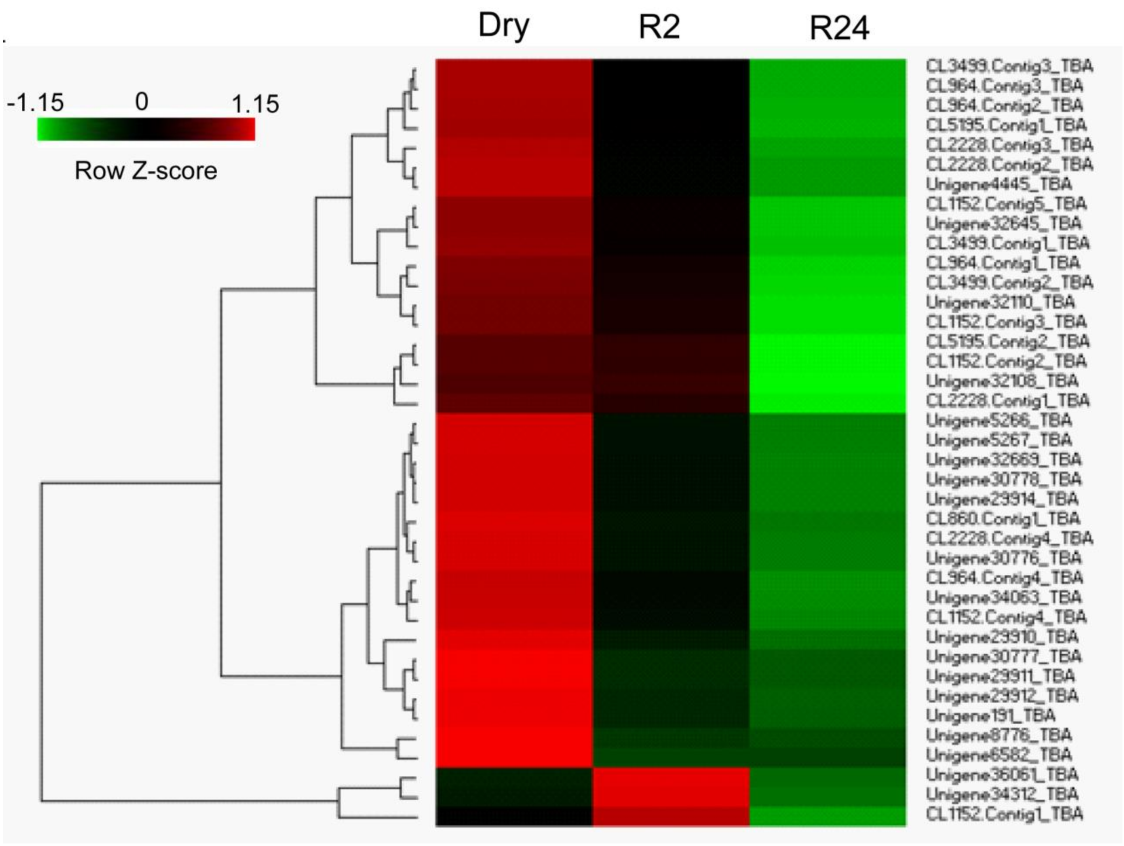

Figure S10 - Hierarchical cluster analyses of the putative early light induced proteins following rehydration. Putative Elips were identified from the transcriptome annotation, each column represents an experimental sample (i.e. Dry, R2 and R24) and each row represents a *B. argenteum* TAC. Expression differences are depicted as normalized Z-scores for each row and shown in different colors, red means high expression and green means low expression.

Table - S3 Primers used for quantitative real-time PCR (qRT-PCR) analysis

Unigene ID	Description	Primer pairs
**CL1684.Contig2_TBA**	Heat shock protein 70	S: CCAGGGCAACCGTATCAC<br>A: CTTCGTCTCGCCATCCTT
**Unigene32542_TBA**	ALDH21	S: TCTGGGAAGCCTATCAAAG<br>A: CCAATCGGAAACTTTCTAAC
**Unigene32354_TBA**	ERF transcription factor	S: AGATTGCGCCGTTCGTCAAG<br>A: GCGGGTCGGTAATATGCTC
**Unigene1001_TBA**	NAC transcription	S: GGAGAAGGGATACTGGAAAG<br>A: CCGTTGTAGTGACTGGGAC
**Unigene26688_TBA**	GRAS transcription factor, DELLA protein	S: GCACGAGAAACTGAACC<br>A: TCTGCTGGGCGAAGTAG
**Unigene25300_TBA**	Nin-like transcription factor	S: ACTCCATTTACCCTCACC<br>A: TCCGCTATACCTAACCTG
**CL2407.Contig1_TBA**	TrDr3 homolog	S: ATTGTGGTGCCGCTCTTT<br>A: AGCATCGTGGGATTAGGAAC
**CL233.Contig1_TBA**	Rehydrin, Tr288 homolog	S: ACGGAGCAGTTCCATTAG

		A: AGCGTCTTTCGCCTTGT
**Unigene32501_TBA**	Rehydrin, Tr155 homolog	S: TTACAGCGTCGCCACTCCT<br>A: GCCATTGCCTGCGTTCTCA
**Unigene32407_TBA**	Dehydrin, LEA protein	S: CTCGTGAGCAGACCCAGTT<br>A: GCAAGGCACAGTCTCAACG
**Unigene31763_TBA**	AP2 transcription factor	S: CATAATGGTCGGTGGGAAGC<br>A: TGCCGTTGTAAGTCTTTGCTG
**Unigene5266_TBA**	ELIPa, Early light induced protein	S: TCTGTCAAGGCTATTGCTGG<br>A: TCTGGCTCACGCTTCGTAC
**CL1148.Contig1_TBA**	Actin*	S: GAGCGGTCTTTCCGAGTAT<br>A: CCATGTCGTCCCAATTAGTC
**CL504.Contig8_TBA**	Tubulin*	S: ATCACGGAGTTCCAGACCAA<br>A: CCACTGCCGCATTCACAT

*used as internal reference gene.
